# Systematic Review and Network Meta-Analysis: Comparative Efficacy and Safety of Biosimilars, Biologics and JAK1 Inhibitors for Active Crohn Disease

**DOI:** 10.3389/fphar.2021.655865

**Published:** 2021-04-14

**Authors:** Guozhi Wu, Yuan Yang, Min Liu, Yuping Wang, Qinghong Guo

**Affiliations:** ^1^ The First Clinical Medical College, Lanzhou University, Lanzhou, China; ^2^ Department of Gastroenterology, The First Hospital of Lanzhou University, Lanzhou, China; ^3^ Gansu Key Laboratory of Gastroenterology, Lanzhou University, Lanzhou, China

**Keywords:** biosimilar, biologics, JAK inhibitors, Crohn disease, network meta analysis

## Abstract

**Background:** Crohn disease (CD) is a chronic inflammatory disease that affects quality of life. There are several drugs available for the treatment of CD, but their relative efficacy is unknown due to a lack of high-quality head-to-head randomized controlled trials.

**Aim:** To perform a mixed comparison of the efficacy and safety of biosimilars, biologics and JAK1 inhibitors for CD.

**Methods:** We searched PubMed, Web of Science, embase and the Cochrane Library for randomized controlled trials (RCTs) up to Dec. 28, 2020. Only RCTs that compared the efficacy or safety of biosimilars, biologics and JAK1 inhibitors with placebo or another active agent for CD were included in the comparative analysis. Efficacy outcomes were the induction of remission, maintenance of remission and steroid-free remission, and safety outcomes were serious adverse events (AEs) and infections. The Bayesian method was utilized to compare the treatments. The registration number is CRD42020187807.

**Results:** Twenty-eight studies and 29 RCTs were identified in our systematic review. The network meta-analysis demonstrated that infliximab and adalimumab were superior to certolizumab pegol (OR 2.44, 95% CI 1.35–4.97; OR 2.96, 95% CI 1.57–5.40, respectively) and tofacitinib (OR 2.55, 95% CI 1.27–5.97; OR 3.10, 95% CI 1.47–6.52, respectively) and revealed the superiority of CT-P13 compared with placebo (OR 2.90, 95% CI 1.31–7.59) for the induction of remission. Infliximab (OR 7.49, 95% CI 1.85–34.77), adalimumab (OR 10.76, 95% CI 2.61–52.35), certolizumab pegol (OR 4.41, 95% CI 1.10–21.08), vedolizumab (OR 4.99, 95% CI 1.19–25.54) and CT-P13 (OR 10.93, 95% CI 2.10–64.37) were superior to filgotinib for the maintenance of remission. Moreover, infliximab (OR 3.80, 95% CI 1.49–10.23), adalimumab (OR 4.86, 95% CI 1.43–16.95), vedolizumab (OR 2.48, 95% CI 1.21–6.52) and CT-P13 (OR 5.15, 95% CI 1.05–27.58) were superior to placebo for steroid-free remission. Among all treatments, adalimumab ranked highest for the induction of remission, and CT-P13 ranked highest for the maintenance of remission and steroid-free remission.

**Conclusion:** CT-P13 was more efficacious than numerous biological agents and JAK1 inhibitors and should be recommended for the treatment of CD. Further head-to-head RCTs are warranted to compare these drugs.

## Introduction

Crohn disease (CD) is a common chronic inflammatory disease with an increasing prevalence and financial burden in recent decades (Ng et al., 2013; Aniwan et al., 2017). With the discovery of novel drug targets, various biologics, such as TNF-α antagonists, integrin and IL-12/23 inhibitors, have been found to have superior therapeutic effectiveness. However, the extensive clinical use of these biological agents is limited due to their AEs and high costs (Odes, 2008; Van Der Valk et al., 2014; Péntek et al., 2017). Therefore, numerous biosimilars are expected to be promising therapies. CT-P13, an IgG1 chimeric human-murine monoclonal antibody biosimilar with the same amino acid sequence as infliximab, has been approved by the US Food and Drug Administration (FDA) for CD (Administration. U. S. F. a. D., 2018). It plays an anti-inflammatory role through the binding of tumor necrosis factor and Fc receptors, the neutralization of tumor necrosis factor, and *in vitro* cytotoxicity (Gabbani et al., 2017). Moreover, some JAK1 inhibitors (tofacitinib, filgotinib, upadacitinib), as oral low-molecular-weight products that affect intracellular molecules involved in signaling of various cytokines, growth factors, and hormones (Schwartz et al., 2016), have also been confirmed to be effective for clinical or endoscopic remission; however, these therapies are also associated with an increased risk of infections (Olivera et al., 2017; Ma et al., 2019). Previous studies have compared the effectiveness of immunosuppressive agents and biological agents, demonstrating the superiority of TNF-α combined with immunosuppressants (Hazlewood et al., 2015). However, many of the RCTs included in these studies had vague definitions of disease activity and primary outcome measures, which might have resulted in an inevitable risk of bias due to clinical heterogeneity. Subsequently, some studies compared biological agents and distinguished between first-line and second-line treatments (Miligkos et al., 2016; Singh et al., 2018). The authors concluded that ustekinumab and vedolizumab were effective for the induction of clinical remission in biologic-naïve patients, although the two were inferior to infliximab and adalimumab. However, the results were inconsistent due to methodological limitations, and studies with more reliable statistical methods are needed to verify the outcomes. With reference to these published articles, we found few studies assessing the effectiveness of biosimilars, biologic agents and small molecule inhibitors. Thus, we conducted a network meta-analysis to compare these therapies based on direct and indirect evidence.

## Methods

### Protocol

A protocol was registered with the International Prospective Register of Systematic Reviews (PROSPERO, www.crd.york.ac.uk/prospero/). The registration number is CRD42020187807.

### Literature Retrieval Strategy

We searched PubMed, Web of Science, Embase, the Cochrane Library and a database for the registration of clinical trials (www.clinicaltrials.gov) for randomized controlled trials (RCTs), with a deadline of Dec. 28, 2020. In addition, the references of articles were manually reviewed to identify uncompleted clinical trials. MeSH/Emtree words, combined with free words, were used for the literature search (the specific search strategy is shown in the Supplementary Material). No limitations were placed on geographic area or language in the literature search process.

### Selection Criteria

Two authors (Guozhi Wu and Yuan Yang) independently selected eligible studies by reading the full text. Discrepancies encountered during studied selection were resolved by negotiating with a third author (Qinghong Guo). The selection criteria were in strict accordance with the PICOS (patients, intervention, comparators, outcomes, study designs) principle: P: patients with active CD (CDAI 220–450); I and C: infliximab, adalimumab, certolizumab, vedolizumab, ustekinumab, tofacitinib, filgotinib, upadacitinib, CT-P13, and placebo; O: outcomes of interest, including 1) primary efficacy data, such as induction of remission and maintenance of remission [defined as an absolute CDAI <150 points during the induction (<20 weeks) and maintenance (≥20 weeks) phases]; secondary efficacy data, such as steroid-free remission (defined as clinical remission without steroid therapy during the maintenance phase); 2) safety data, such as the proportion of patients with adverse events (AEs) and infections or serious/severe infections; S: randomized controlled studies.

Studies were excluded if they 1) evaluated biologics-failure patients; 2) only assessed the efficacy and safety of therapies for pediatric, elderly, postoperative and fistulizing patients; 3) included other subtypes of inflammatory bowel diseases; 4) were duplicate publications; 5) were reviews, letters, conference abstracts, animal studies, etc. Because there was a lack of RCTs that evaluated the biosimilar SB2, this drug was excluded from this meta-analysis. In addition, studies that could not be compared to others through a common comparator were excluded.

### Risk of Bias Analysis

Risk of bias was evaluated separately by two authors (Guozhi Wu and Yuan Yang) using the Cochrane Collaboration’s risk of bias tool (version 5.1.0) Any disagreement was resolved through negotiation with a third reviewer (Qinghong Guo). Seven domains (selective bias for randomization, selective bias for assignment, performance bias, detection bias, attrition bias, selective reporting bias, other bias) were assessed separately as “low risk”, “unclear risk” or “high risk”.

### Data Extraction

Two authors (Guozhi Wu and Yuan Yang) independently extracted data related to the author, publication date, number of patents, country, treatment drugs, treatment regimens, definition of outcomes, duration of follow-up, baseline severity of disease, concomitant therapies, biological agent exposure history, efficacy and safety of the included studies. Discrepancies were resolved by a third author (Qinghong Guo) if there was any uncertainty regarding the data extraction.

For trials that assessed different doses of drugs, we combined these subgroups; for crossover trials, we only extracted the data from before the crossover. If there were multiple outcomes at different times, we extracted the earliest result for the induction phase (for example, week six instead of 8) and the latest result for the maintenance phase (week 54 instead of 30).

### Data Analysis and Mixed Treatment Comparison

We analyzed the efficacy and safety data for treatments separately and adopted odds ratios (ORs) with 95% CI to express the effect estimate. A random-effect Bayesian network meta-analysis was conducted to perform pairwise comparisons of the efficacy and safety of therapies in the induction and maintenance phases. Using a full Bayesian evidence network, all indirect comparisons were taken into account to arrive at a single integrated estimate of the effect of all included treatments based on all included studies. However, even with a consistent set of relative effect estimates, it may still be difficult to draw conclusions from a potentially large set of treatments. Luckily, the Bayesian approach allowed us to estimate the probability that, given the priors and the data, each of the treatments will be the best, the second best, etc. This information is provided below in the rank probability plot. In the Bayesian model, 4 chains were run with 20,000 tuning iterations and 200,000 simulation iterations. Convergence was assessed using the Brooks-Gelman-Rubin method. This method compares within-chain and between-chain variance to calculate the potential scale reduction factor (PSRF). A PSRF close to one indicates that approximate convergence has been reached. Moreover, publication bias was evaluated by drawing funnel plots and checking for asymmetry. All analyses were conducted in STATA (version 16.0), WinBUGS (version 1.4.3) and R statistical software (version 4.0.3).

### Sensitivity Analyses

For induction of remission: 1) only trials that assessed efficacy for biologics-naïve patients were included; 2) trials identified as having a high risk of bias were excluded. For maintenance of remission, sensitivity analyses were performed by excluding treat-through trials (i.e., those that continued to treat regardless of response or nonresponse after induction therapy).

## Results

### Literature Search and Risk of Bias Assessment

**FIGURE 1:**
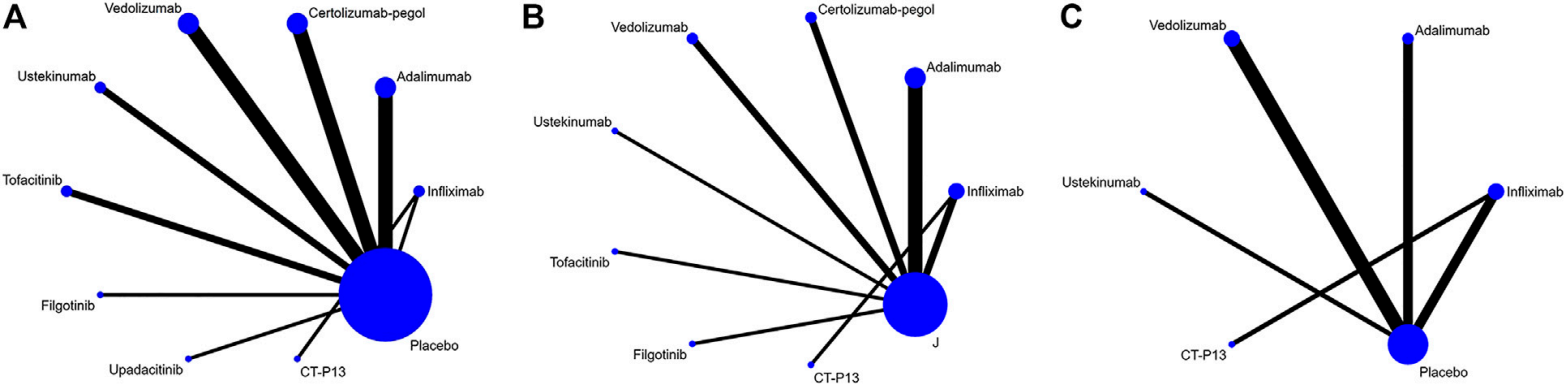
Network plot for **(A)** induction of remission **(B)** maintenance of remission **(C)** steroid-free remission.

We initially retrieved 2,377 studies from PubMed, 1,463 from the Cochrane Library, 1,644 from Web of Science and 7,873 from Embase. After screening (the specific literature screening process is shown in Supplementary Figure S1), we identified 28 studies and 29 trials in our analyses. Seventeen trials evaluated TNF-α inhibitors (infliximab, adalimumab, and certolizumab pegol), 4 trials evaluated integrin monoclonal antibody (vedolizumab), 3 trials evaluated IL-12/IL-23 monoclonal antibody (ustekinumab), 4 trials evaluated JAK1 inhibitors (tofacitinib, filgotinib and upadacitinib) and 1 trial compared an infliximab biosimilar (CT-P13) to infliximab. Induction of remission was assessed in 21 trials, maintenance of remission in 15 trials and steroid-free remission in 9 trials. Safety data were provided in all trials. Due to a lack of relevant data on upadacitinib, we had to abandon the assessment of its efficacy and safety in the maintenance phase. Of the maintenance trials, 2 were treat-through trials, and 13 only included responders to induction therapy. The characteristics of the included studies are summarized in Table 1. Network plots and funnel plots are showed in Figure 1 and Supplementary Figures S3, S4. The risk-of-bias assessment is shown in Supplementary Figure S2. Of the total group of 27 studies, Schreiber et al., 2005 was judged as high risk due to the absence of double-blinding.

**TABLE 1:** Characteristics of studies included in the comparison.

Study	Trial design	Country; number of sites	Interventions	Number of patients	Definition of outcomes	Time points of clinical outcomes, week	Severity of CD at randomization	Concomitant treatments	Prior biologics
Infliximab									
Targan et al. (1997)	Induction	North America and Europe; 18	Infliximab 5, 10, or 20 mg/kg IV at week 0; placebo	108	CDAI<150	4 and 12	CDAI (220–400)	Steroids, 59%; immunosuppressants, 37%	0%
Lemann et al. (2006)	Treat-through	France; 20	Infliximab 5 mg/kg IV at weeks 0, 2, and 6 + azathioprine or 6-MP; azathioprine (2–3 mg/kg) or 6-MP (1–1.5 mg/kg) + placebo	115	Steroid-free remission	12 and 24	Patients with active disease despite 6 months of steroids	All patients on tapering dose of steroids; immunosuppressants other than azathioprine/6 MP were not allowed	0%
Hanauer et al. 2002 (ACCENT I)	Maintenance	North America, Europe, and Israel; 55	Infliximab 5 or 10 mg/kg IV every 8 weeks after induction; placebo	335	CDAI<150 and steroid-free remission	30 and 54	CDAI decrease ≥70 points and 25% reduction from baseline (220–400) after induction therapy	Steroids, 52%; immunosuppressants, 27%	0%
Rutgeerts et al. (1999)	Maintenance	North America and Europe; 17	Infliximab 10 mg/kg every 8 weeks IV after single-dose induction therapy; placebo	73	CDAI<150	44	CDAI decrease from baseline ≥70 points after induction therapy	NA	0%
Colombel et al. (2010) (SONIC)	Treat-through (with induction data)	North America, Europe, and Israel; 92	Infliximab 5 mg/kg IV at weeks 0, 2, 6, 14, and 22; azathioprine 2.5 mg/kg/day: combination therapy (infliximab + azathioprine)	508	CDAI<150 and steroid-free remission	10 and 26	CDAI (220–450)	Steroids, 87%; immunosuppressants, 0%	0%
Adalimumab									
Hanauer et al. (2006) (CLASSIC-I)	Induction	North America and Europe; 55	Adalimumab 40 mg/20 mg, 40 mg/80 mg, or 80 mg/160 mg SC at weeks 0 and 2; placebo	299	CDAI<150	4	CDAI (220–450)	Steroids, 33%; immunosuppressants, 29%	0%
Sandborn et al. (2007a) (Gain)	Induction	North America and Europe; 52	Adalimumab 160/80 mg SC at weeks 0 and 2; placebo	325	CDAI<150	4	CDAI (220–450)	Steroids, 39%; immunosuppressants, 49%	100%
Watanabe et al. (2012)	Induction	Japan; 2	Adalimumab 160/80 mg or 80/40 mg SC at weeks 0 and 2; placebo	90	CDAI<150	4	CDAI (220–450)	Steroids, 21%; immunosuppressants, 32%	58%
Chen et al. (2020)	Induction	China; 15	Adalimumab 160 mg at week 0, 80 mg at week 2; placebo	205	CDAI<150	4	CDAI (220–450)	Mandatory corticosteroid dose tapering; doses of immunosuppressants, aminosalicylates, and antibiotics remained stable	0%
Rutgeerts et al. (2012) (EXTEND)	Maintenance	North America and Europe; 19	Adalimumab 40 mg SC EOW; placebo	129	CDAI<150	52	CDAI decrease from baseline ≥70 points after induction therapy	Steroids, 26%; immunosuppressants,41%	52%
Colombel et al. (2007) (CHARM)	Maintenance	North America, Europe, South Africa, Australia; 92	Adalimumab 40 mg weekly SC, or 40 mg SC EOW after induction; placebo	778	CDAI<150 and rate of complete discontinuation of steroids	26 and 56	CDAI decrease ≥70 points from baseline after induction therapy	Steroids, 44%; immunosuppressants, 47%	50%
Sandborn et al. (2007b) (CLASSICII)	Maintenance	North America, Europe; 53	Adalimumab 40 mg weekly SC, or 40 mg SC EOW after induction; placebo	55	CDAI<150 and rate of completely discontinuation of steroids	56	CDAI <150 after induction therapy	Steroids, 49%; immunosuppressants, 22%	0%
Watanabe et al. (2012)	Maintenance	Japan; 2	Adalimumab 40 mg SC EOW; placebo	43	CDAI<150	52	CDAI decrease ≥70 points from baseline after 4 weeks of induction therapy	Steroids, 16%; immunosuppressants, 36%	54%
Certolizumab									
Sandborn et al. (2011)	Induction	Multinational; 120	Certolizumab 400 mg SC at weeks 0, 2, and 4; placebo	439	CDAI<150	6	CDAI (220–450)	Steroids, 45%; immunosuppressants, 33%	0%
Schreiber et al. (2005)	Induction	North America, Europe, and South Africa; 58	Certolizumab 100, 200, or 400 mg SC at weeks 0, 4, and 8; Placebo	292	CDAI<150	12	CDAI (220–450)	Steroids, 36%; immunosuppressants, 37%	22%
Winter et al. (2004)	Induction	Israel, Europe, and South Africa; 24	Certolizumab 5, 10, or 20 mg/kg IV at week 0; placebo	92	CDAI<150	4	CDAI (220–450)	Steroids, 28%; immunosuppressants, 45%	24%
Sandborn et al. (2007c) (PRECISE1)	Treat-through (with induction data)	Multinational; 171	Certolizumab 400 mg SC at weeks 0, 2, and 4, then every 4 weeks; placebo	662	CDAI<150	26	CDAI (220–450)	Steroids, 39%; immunosuppressants, 37%	28%
Schreiber et al. (2007) (PRECISE2)	Maintenance	Multinational; 147	Certolizumab 400 mg SC every 4 weeks after induction; placebo	428	CDAI<150	26	CDAI decrease ≥100 points from baseline after induction therapy	Steroids, 36%; immunosuppressants, 40%	24%
Vedolizumab									
Feagan et al. (2008)	Induction	Canada; 21	Vedolizumab 0.5 or 2 mg/kg IV at weeks 0 and 4; placebo	185	CDAI<150	8	CDAI (220–450)	Steroids, 0%; immunosuppressants, 0%	0%
Sandborn et al. (2013) (GEMINI2a)	Induction	Multinational; 285	Vedolizumab 300 mg IV at weeks 0 and 2; placebo	368	CDAI<150	6	CDAI (220–450)	Steroids, 34%; immunosuppressants, 17%	62%
Sands et al. (2014) (GEMINI3)	Induction	Multinational; 107	Vedolizumab 300 mg IV at weeks 0, 2, and 6; placebo	101	CDAI<150	6	CDAI (220–450)	Steroids, 22%; immunosuppressants, 30%	0%
Watanabe et al. (2020)	Induction	Japan; 71	Vedolizumab 300 mg IV at weeks 0, 2, and 6; placebo	157	CDAI<150	10	CDAI (220–450)	Steroids, 25%; immunosuppressants, 48%	78%
Sandborn et al. (2013) (GEMINI2a)	Maintenance	Multinational; 285	Vedolizumab 300 mg IV every 4 or every 8 weeks; placebo	461	CDAI<150 and steroid-free remission	52	CDAI<150 after induction therapy	Steroids, 36%; immunosuppressants, 17%	54%
Watanabe et al. (2020)	Maintenance	Japan; 71	Vedolizumab 300 mg IV at week 14, then every 8 weeks until week 54; placebo	24	CDAI<150	60	CDAI<150 after induction therapy	Steroids, 33%; immunosuppressants, 63%	62.50%
Ustekinumab									
Sandborn et al. (2008)	Crossover (with induction data)	NA	Placebo at weeks 0, 1, 2, and 3, then ustekinumab 90 mg IH at weeks 8, 9, 10, and 11; ustekinumab 90 mg IH at weeks 0, 1, 2, and 3, then placebo at weeks 8, 9, 10, and 11; placebo at weeks 0, then ustekinumab 4.5 mg/kg IV at weeks 8; ustekinumab 4.5 mg/kg IV at weeks 0, then placebo at week 8	104	CDAI<150	6	CDAI (220–450)	Steroids, 32%; immunosuppressants, 34%	47%
Feagan et al. (2016) (UNITI-2)	Induction	Multinational; 175	Ustekinumab 130 mg IV at week 0; placebo	628	CDAI<150	6	CDAI (220–450)	Steroids, 39%; immunosuppressants, 35%	31%
Feagan et al. (2016) (IM-UNITI)	Maintenance	Multinational; 260	Ustekinumab 90 mg IV every 8 weeks through week 40; ustekinumab 90 mg IV every 12 weeks through week 40; placebo	397	CDAI<150 and steroid-free remission	52	CDAI decrease ≥100 points from baseline after induction therapy	Steroids, 45.6%; immunosuppressants, 36%	39.50%
Tofacitinib									
Sandborn et al. (2014)	Induction	Multinational; 48	Tofacitinib 1 mg twice daily, 5 mg twice daily, 15 mg twice daily; placebo twice daily	139	CDAI<150	4	CDAI (220–450)	Immunosuppressants, 1%	7%
Panes et al. (2017)	Induction	Multinational; 80	Tofacitinib 5 mg twice daily, 10 mg twice daily, 15 mg twice daily; placebo twice daily	280	CDAI<150	8	CDAI (220–450)	Steroids, 35.5%	77%
Panes et al. (2017)	Maintenance	Multinational; 80	Tofacitinib 5 twice mg daily, 10 mg twice daily; placebo twice daily	180	CDAI<150	26	CDAI decrease ≥100 points from baseline or CDAI<150 after induction therapy	Steroids, 33%	75%
Filgotinib									
Vermeire et al. (2017) (FITZROY)	Induction	Multinational; 52	Filgotinib 200 mg once a day; placebo	174	CDAI<150	10	CDAI (220–450)	Steroids, 51%	58%
Vermeire et al. (2017) (FITZROY)	Maintenance	Multinational; 52	Filgotinib 200 mg once a day, 100 mg once a day; placebo	74	CDAI<150	20	CDAI decrease ≥100 points from baseline after induction therapy	Forced steroid reduction after week 10	NA
Upadacitinib									
Sandborn et al. (2020) (CELEST)	Induction	Multinational; 93	Upadacitinib 3 mg, 6 mg, 12 mg, 24 mg twice daily or 24 mg once daily; placebo	220	CDAI<150	16	CDAI (220–450)	Steroids, 44%	58%
CT-P13									
Ye et al. (2019)	Induction	Multinational; 58	CT-P13 5 mg/kg IV at weeks 0, 2, and 6; infliximab 5 mg/kg IV at weeks 0, 2, and 6	220	CDAI<150	6	CDAI (220–450)	Steroids, 32%	0%
Ye et al. (2019)	Maintenance	Multinational; 58	CT-P13 5 mg/kg IV at week 14 and every eight weeks up to week 54; infliximab 5 mg/kg IV at week 14 and every eight weeks up to week 54	110 and 92	CDAI<150 and steroid-free remission	54	Reduction of at least 70 points from the baseline CDAI (220–450) after induction therapy	NA	0%

### Induction of Remission

**FIGURE 2:**
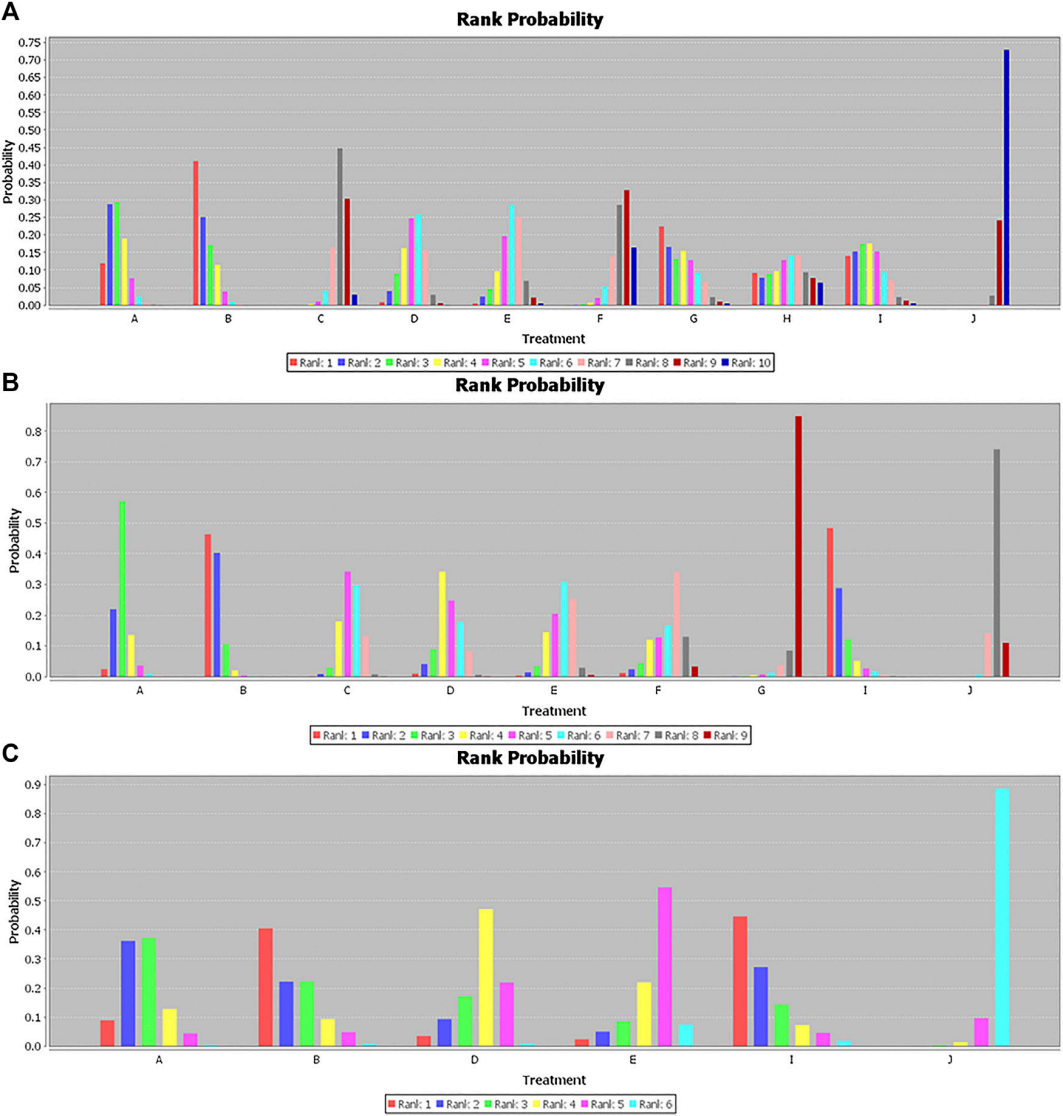
Rank probability for **(A)** induction of remission. **(B)** Maintenance of remission. **(C)** Steroid-free remission. A, Infliximab; B, Adalimumab; C, Certolizumab pegol; D, Vedolizumab; E, Ustekinumab; F, Tofacitinib; G, Filgotinib; H, Upadacitinib; I, CT-P13; J, Placebo.

In the Bayesian network meta-analysis, infliximab, adalimumab, vedolizumab, ustekinumab, and filgotinib showed a statistically significant effect on the induction of remission compared to placebo. The difference was not statistically significant for certolizumab pegol, but the trend (OR 1.35, 95% CI 0.96–1.91) favored its effect on the induction of remission (Table 2). Of note, we observed the superiority of infliximab compared with certolizumab pegol and tofacitinib (OR 2.44, 95% CI 1.35–4.97; OR 2.55, 95% CI 1.27–5.97, respectively), the superiority of adalimumab compared with certolizumab pegol and tofacitinib (OR 2.94, 95% CI 1.63–5.22; OR 3.10, 95% CI 1.47–6.52, respectively) and the superiority of CT-P13 compared with placebo (OR 2.90, 95% CI 1.31–7.59). There was no significant difference between biological agents and the biosimilar CT-P13 in the induction of remission (Table 2). The rank probability result favors the superiority of adalimumab over other interventions for the induction of remission (Figure 2A).

**TABLE 2:** Pairwise comparisons of induction of remission.

Intervention	Odds ratios (95% CI)								
Comparator	Infliximab	Adalimumab	Certolizumab	Vedolizumab	Ustekinumab	Tofacitinib	Filgotinib	Upadacitinib	CT-P13
Adalimumab	0.84 (0.43, 1.83)	—							
Certolizumab	**2.44 (1.35, 4.97)**	**2.94 (1.63, 5.22)**	—						
Vedolizumab	1.47 (0.73, 3.12)	1.74 (0.88, 3.29)	0.60 (0.33, 1.04)	—					
Ustekinumab	1.62 (0.81, 3.68)	1.92 (0.98, 3.85)	0.66 (0.37, 1.24)	1.11 (0.57, 2.27)	—				
Tofacitinib	**2.55 (1.27, 5.97)**	**3.10 (1.47, 6.52)**	1.05 (0.56, 2.02)	1.76 (0.87, 3.75)	1.58 (0.76, 3.41)	—			
Filgotinib	1.07 (0.38, 3.16)	1.29 (0.45, 3.32)	0.43 (0.16, 1.08)	0.74 (0.26, 1.96)	0.65 (0.23, 1.74)	0.41 (0.15, 1.13)	—		
Upadacitinib	1.54 (0.47, 5.33)	1.87 (0.55, 5.59)	0.63 (0.20, 1.88)	1.06 (0.32, 3.25)	0.95 (0.29, 2.98)	0.59 (0.17, 1.97)	1.45 (0.36, 6.18)	—	
CT-P13	1.13 (0.56, 2.17)	1.35 (0.47, 3.34)	0.46 (0.17, 1.11)	0.78 (0.27, 1.99)	0.69 (0.23, 1.87)	0.44 (0.15, 1.13)	1.06 (0.30, 3.57)	0.74 (0.18, 2.89)	—
Placebo	**3.30 (2.01, 6.17)**	**3.93 (2.47, 6.33)**	1.35 (0.96, 1.91)	**2.26 (1.45, 3.65)**	**2.02 (1.23, 3.39)**	1.28 (0.73, 2.21)	**3.08 (1.34, 7.86)**	2.13 (0.76, 6.38)	**2.90 (1.31, 7.59)**

Bold type represents statistically significant superiority/inferiority of the intervention over the comparator.

### Maintenance of Remission

Infliximab, adalimumab, certolizumab pegol, vedolizumab, and ustekinumab all demonstrated a statistically significant effect on the maintenance of remission compared to placebo (Table 3). We observed that CT-P13 also showed a better effect on the maintenance of remission than placebo (OR 5.01, 95% CI 1.86–13.76) based on the network meta-analysis. Moreover, our study showed that infliximab, adalimumab, certolizumab pegol, vedolizumab, and CT-P13 were superior to filgotinib (OR 7.49, 95% CI 1.85–34.77; OR 10.76, 95% CI 2.61–52.35; OR 4.41, 95% CI 1.10–21.08; OR 4.99, 95% CI 1.19–25.54; OR 10.93, 95% CI 2.10–64.37, respectively). In addition, adalimumab had a statistically significant effect on the maintenance of remission compared to certolizumab, ustekinumab, tofacitinib, and filgotinib (OR 2.46, 95% CI 1.27–4.79; OR 2.67, 95% CI 1.19–6.07; OR 3.03, 95% CI 1.01–8.63, OR 10.76 95% CI 2.61–52.35, respectively) and showed numerically rather than statistically significant trends toward superiority compared to vedolizumab (OR 2.16, 95% CI 0.97–4.67) (Table 3). The rank probability results favored the superiority of CT-P13 and adalimumab over other interventions for the maintenance of remission (Figure 2B).

**TABLE 3:** Pairwise comparisons of maintenance of remission.

Intervention	Odds ratios (95% CI)							
Comparator	Infliximab	Adalimumab	Certolizumab	Vedolizumab	Ustekinumab	Tofacitinib	Filgotinib	CT-P13
Adalimumab	0.69 (0.35, 1.38)	—						
Certolizumab	1.69 (0.91, 3.21)	**2.46 (1.27, 4.79)**	—					
Vedolizumab	1.50 (0.69, 3.11)	2.16 (0.97, 4.67)	0.88 (0.41, 1.80)	—				
Ustekinumab	1.84 (0.85, 4.11)	**2.67 (1.19, 6.07)**	1.08 (0.50, 2.37)	1.24 (0.52, 3.09)	—			
Tofacitinib	2.09 (0.71, 5.83)	**3.03 (1.01, 8.63)**	1.25 (0.42, 3.37)	1.42 (0.45, 4.24)	1.13 (0.35, 3.52)	—		
Filgotinib	**7.49 (1.85, 34.77)**	**10.76 (2.61, 52.35)**	**4.41 (1.10, 21.08)**	**4.99 (1.19, 25.54)**	4.06 (0.92, 20.40)	3.60 (0.70, 22.31)	—	
CT-P13	0.68 (0.28, 1.70)	1.00 (0.33, 3.09)	0.41 (0.14, 1.20)	0.46 (0.15, 1.50)	0.38 (0.11, 1.23)	0.34 (0.09, 1.33)	0.09 (0.02, 0.48)	—
Placebo	**3.44 (2.24, 5.52)**	**5.02 (3.03, 8.34)**	**2.04 (1.34, 3.15)**	**2.30 (1.31, 4.40)**	**1.87 (1.00, 3.57)**	1.65 (0.66, 4.45)	0.47 (0.10, 1.76)	**5.01 (1.86, 13.76)**

Bold type represents statistically significant superiority/inferiority for the intervention over the comparator.

### Steroid-Free Remission

Because of its sparse data on steroid-free remission, we excluded JAK1 inhibitors. With the exception of ustekinumab (OR 1.91, 95% CI 0.59, 6.20), we observed statistically significant differences in the effects of infliximab, adalimumab, certolizumab pegol, vedolizumab, and CT-P13 over placebo for steroid-free remission (OR 3.80, 95% CI 1.49–10.23; OR 4.86, 95% CI 1.43–16.95; OR 2.48, 95% CI 1.21–6.52; OR 5.15, 95% CI 1.05–27.58, respectively), but there was no statistically significant difference among these six treatments (Table 4). The rank probability results favored the superiority of CT-P13 over other treatments for steroid-free remission (Figure 2C).

**TABLE 4:** Pairwise comparisons of steroid-free remission.

Intervention	Odds ratios (95% CI)				
Comparator	Infliximab	Adalimumab	Vedolizumab	Ustekinumab	CT-P13
Adalimumab	0.79 (0.17, 4.00)	—			
Vedolizumab	1.53 (0.40, 5.03)	1.95 (0.41, 7.91)	—		
Ustekinumab	1.98 (0.46, 9.34)	2.56 (0.48, 13.04)	1.29 (0.35, 6.07)	—	
CT-P13	0.74 (0.20, 2.71)	0.94 (0.12, 6.85)	0.48 (0.08, 3.24)	0.37 (0.05, 2.51)	—
Placebo	**3.80 (1.49, 10.23)**	**4.86 (1.43, 16.95)**	**2.48 (1.21, 6.52)**	1.91 (0.59, 6.20)	**5.15 (1.05, 27.58)**

Bold type represents statistically significant superiority/inferiority for the intervention over the comparator.

### Safety Data

No statistically significant difference was observed regarding the rate of AEs and infections or serious/severe infections in either the induction or maintenance phases among the evaluated treatments, except between ustekinumab and upadacitinib (OR 0.30, 95% CI 0.09–0.95) (Supplementary Tables S2, S4). Nevertheless, the rank probability results demonstrated that filgotinib and upadacitinib seemed to increase the probability of AEs in the induction phase. Total infections or serious/severe infections were more likely with upadacitinib but less likely with tofacitinib in the induction phase (Supplementary Table S3). In the maintenance phase, tofacitinib was associated with a higher probability of infections or serious/severe infections (Supplementary Table S5). Compared with JAK1 inhibitors, the biosimilar CT-P13 and biologics showed a lower likelihood of AEs and infectious events (Supplementary Table S5).

### Sensitivity Analyses

For the induction of remission, the results were similar when studies with a high risk of bias were excluded. When only trials that evaluated biologics-naïve patients were included, only infliximab and adalimumab showed statistically significant superiority for the induction of remission. For maintenance of remission, the exclusion of treat-through trials contributed to the loss of statistically significant superiority of adalimumab relative to certolizumab pegol (data not shown).

## Discussion

Although biologics have developed rapidly and have significantly improved clinical and endoscopic outcomes, their high costs and risk of infections and malignancies limit their application (Bonovas et al., 2016). Recently, new therapies such as biosimilars and small molecular inhibitors have shown promise for application in clinical practice (Brodszky et al., 2014; Navarro et al., 2020; Tian et al., 2020; Vellopoulou et al., 2020). Nevertheless, only limited studies available assessing the effectiveness and tolerance of these drugs are available. In particular, no direct comparisons of these novel treatments with traditional biological agents were found. Hence, a Bayesian network meta-analysis was conducted to evaluate the efficacy and safety of biologics, biosimilar agents and JAK1 inhibitors for the induction and maintenance of remission in CD.

In this meta-analysis, traditional TNF-α antagonists and the biosimilar CT-P13 demonstrated similar effectiveness and favorable tolerance. The superiority of infliximab and adalimumab was shown for the induction of remission, consistent with a previous comparative analysis (Hazlewood et al., 2015). However, there was no significant difference between infliximab and certolizumab pegol (OR 2.1, 95% CI 0.98–5.5) in this study. Considering that this comparative study defined the primary outcome as steroid-free clinical remission, possible clinical heterogeneity may have resulted in bias. Therefore, steroid-free remission was listed independently in our study rather than being included with clinical remission (this definition is noted in the *Method section*). In addition, CT-P13 showed no obvious differences from other therapies for the induction of remission, a result that has not been confirmed in the past few years. A large-scale randomized, placebo-controlled, double-blind phase 3 study aimed at evaluating the efficacy and safety of CT-P13 is ongoing (Celltrion, 2019), and it is hoped that the results will provide further support for our findings. Moreover, our analyses indicated that there were higher probabilities of AEs and infectious AEs with filgotinib and upadacitinib. However, the results should be interpreted cautiously because insufficient real-life data and phase 3 RCT evidence is available for evaluating their safety profile. Therefore, in the clinical application of novel JAK1 inhibitors, efficacy and AEs should be carefully weighed.

In the maintenance phase, we observed the inferiority of filgotinib to biologics and biosimilars, which might limit its clinical application despite the lack of a significant increase in AEs and infectious AEs. The ongoing phase 3 clinical trial may expand the sample size and further confirm the efficacy and safety of this agent (Sciences and Nv, 2016). In addition, although our analysis showed that adalimumab had greater efficacy than tofacitinib, no difference was found between tofacitinib and other biologics. Additionally, we could not neglect the higher risk of infectious AEs with tofacitinib in the maintenance period, as shown in the SUCRA results (rank probability). Nevertheless, in addition to safety considerations, decisions to prescribe tofacitinib should not ignore its lower costs (Van Der Valk et al., 2014) compared with biologics. Furthermore, it is worth noting that no significant difference in efficacy and safety was observed between CT-P13 and other therapies; however, SUCRA favored the effectiveness and tolerance of CT-P13, which provides a direction for further studies of the efficacy of this drug compared to biologics. We believe that with a lower risk of AEs, infections and costs, CT-P13, given its superiority over other agents, might be the primary choice for the maintenance of remission.

Patients with active CD initially use corticosteroids to control their symptoms, but many patients are prone to increased risk of mortality due to resistance to corticosteroids (Lewis et al., 2008; Lewis et al., 2018). Hence, it is worth mentioning that steroid-free remission, as a secondary outcome of efficacy, was assessed in this study. JAK1 inhibitors and certolizumab pegol were excluded from this comparative treatment analysis due to a lack of trials. In the Bayesian model, infliximab, adalimumab, vedolizumab, and CT-P13 showed a statistically significant rate of steroid-free remission compared to placebo. Although no significant difference was observed, CT-P13 and adalimumab ranked highest for steroid-free remission, indicating their superiority over other therapies.

Our sensitivity analyses also revealed the efficacy and safety of infliximab and adalimumab for the induction of remission in biologics-naïve patients. No significant effect was observed for vedolizumab and ustekinumab, in contrast to a previous study (Singh et al., 2018). Because that study conducted a network meta-analysis based on the frequency analysis method, the results should be interpreted with caution. The frequency method relies primarily on the maximum likelihood method for parameter estimation; however, the maximum likelihood function is estimated through continuous iteration, which is prone to instability and biased results. As a result, vedolizumab and ustekinumab could not be included in our analysis of the induction of remission in biologics-naïve patients.

The performance of the novel drug CT-P13 in our study is worthy of mention. Most current RCTs have focused on the comparison of CT-P13 and the innovator, infliximab (Ye et al., 2017; Goll et al., 2019; Ye et al., 2019), and there is little information about the relative efficacy and safety of CT-P13 and other treatments. Our network meta-analysis helped to determine the sequence of prescription for CT-P13 relative to TNF-α inhibitors, JAK1 inhibitors and the negative control (placebo). However, no significant differences among CT-P13, filgotinib and upadacitinib were observed. This result might be explained by the lack of large-scale phase three clinical trials evaluating the latter two agents, which contributed to a wide confidence interval. In summary, our comparative analysis suggests that the decision to prescribe CT-P13 should balance its relative effectiveness against its profile of fewer AEs and lower costs. More cost-effectiveness and RCT studies are needed to determine the prescribing sequence for CT-P13.

There are some limitations in this study. First, only 10 interventional studies were included, and there were few direct comparisons and no closed loops in the network plot. However, the absence of closed loops did not significantly affect our results compared to other network meta-analyses. In addition, the exclusion of treat-through trials contributed to a loss of superiority of adalimumab relative to certolizumab pegol for maintenance of remission. Due to the lack of trials assessing the effectiveness of filgotinib in our analyses, uncertainties regarding the point estimates remain. Similarly, limited data were available on upadacitinib during the maintenance phase. Furthermore, the lack of large-scale phase three clinical trials of JAK1 inhibitors resulted in wide confidence intervals. Finally, real-life data, especially for safety, were not included in our network meta-analysis.

## Conclusion

In this study, we concluded that adalimumab ranked highest for the induction of remission and CT-P13 ranked highest for the maintenance of remission and steroid-free remission. These two drugs should be recommended for active CD. Further head-to-head RCTs are warranted to compare these drugs

## Data Availability

The original contributions presented in the study are included in the article/Supplementary Material, further inquiries can be directed to the corresponding author.
