# Supplementary material for: Systematic Review and Network Meta-Analysis: Comparative Efficacy and Safety of Biosimilars, Biologics and JAK1 Inhibitors for Active Crohn Disease
Source: Front Pharmacol. 2021 Apr 14;12:655865. doi: 10.3389/fphar.2021.655865 (PMC8080031; doi:10.3389/fphar.2021.655865)
Supplement: Supplementary file 1 [file datasheet1.docx]

Supplementary Material

# Search strategy of PubMed

((((((((Crohn Disease[Title/Abstract]) OR (Crohn's Enteritis[Title/Abstract])) OR (Regional Enteritis[Title/Abstract])) OR (Crohn's Disease[Title/Abstract])) OR (Crohns Disease[Title/Abstract])) OR ("Crohn Disease"[Mesh])) AND (((((((((((((((((Tumor Necrosis Factor Inhibitor*[Title/Abstract]) OR (Tumor Necrosis Factor Blocker*[Title/Abstract])) OR (Tumor Necrosis Factor Antagonist*[Title/Abstract])) OR (TNF Inhibitor*[Title/Abstract])) OR (TNF Blocker*[Title/Abstract])) OR (TNF Antagonist*[Title/Abstract])) OR ("Tumor Necrosis Factor Inhibitors"[Mesh])) OR (((Infliximab[Title/Abstract]) OR (Remicade[Title/Abstract])) OR ("Infliximab"[Mesh]))) OR (((Adalimumab[Title/Abstract]) OR (Humira[Title/Abstract])) OR ("Adalimumab"[Mesh]))) OR (((Certolizumab Pegol[Title/Abstract]) OR (Cimzia[Title/Abstract])) OR ("Certolizumab Pegol"[Mesh]))) OR (((Vedolizumab[Title/Abstract]) OR (Entyvio[Title/Abstract])) OR ("vedolizumab" [Supplementary Concept]))) OR (((Ustekinumab[Title/Abstract]) OR (Stelara[Title/Abstract])) OR ("Ustekinumab"[Mesh]))) OR ((((CT-P13[Title/Abstract]) OR (Inflextra[Title/Abstract])) OR (Remsima[Title/Abstract])) OR ("CT-P13" [Supplementary Concept]))) OR (((Janus Kinase Inhibitor*[Title/Abstract]) OR (JAK Inhibitor*[Title/Abstract])) OR ("Janus Kinase Inhibitors"[Mesh]))) OR (((tofacitinib[Title/Abstract]) OR (tasocitinib[Title/Abstract])) OR ("tofacitinib" [Supplementary Concept]))) OR ((GLPG0634[Title/Abstract]) OR (filgotinib[Title/Abstract]))) OR (((upadacitinib[Title/Abstract]) OR (ABT-494[Title/Abstract])) OR ("upadacitinib" [Supplementary Concept])))) AND (("randomized controlled trial"[pt] OR "controlled clinical trial"[pt] OR randomized[tiab] OR placebo[tiab] OR "drug therapy"[sh] OR randomly[tiab] OR trial[tiab] OR groups[tiab]))) NOT ("cohort studies"[mesh] OR "case-control studies"[mesh] OR "comparative study"[pt] OR "risk factors"[mesh] OR "cohort"[tw] OR "compared"[tw] OR "groups"[tw] OR "case control"[tw] OR "multivariate"[tw])

# Search strategy of Embase

#1 'crohn disease'/exp OR 'crohn disease' OR 'crohns enteritis' OR 'regional enteritis' OR 'crohns disease':ti,ab

#2 'tumor necrosis factor inhibitor'/exp OR 'tumor necrosis factor inhibitor' OR 'tumor necrosis factor blocker' OR 'tumor necrosis factor antagonist' OR 'TNF inhibitor' OR 'TNF blocker' OR 'TNF antagonist' OR 'tumor necrosis factor inhibitors' OR 'tumor necrosis factor blockers' OR 'tumor necrosis factor antagonists' OR 'TNF inhibitors' OR 'TNF blockers' OR 'TNF antagonists':ti,ab

#3 'infliximab'/exp OR 'infliximab' OR 'remicade':ti,ab

#4 'adalimumab'/exp OR 'adalimumab' OR 'humira':ti,ab

#5 'certolizumab pegol'/exp OR 'certolizumab pegol' OR 'cimzia':ti,ab

#6 'vedolizumab'/exp OR 'vedolizumab' OR 'entyvio':ti,ab

#7 'ustekinumab'/exp OR 'ustekinumab' OR 'stelara':ti,ab

#8 'ct-p13'/exp OR 'ct-p13' OR 'inflextra' OR 'remsima':ti,ab

#9 'janus kinase inhibitor'/exp OR 'janus kinase inhibitor' OR 'janus kinase inhibitors' OR 'JAK inhibitor' OR 'JAK inhibitors':ti,ab

#10 'tofacitinib'/exp OR 'tofacitinib' OR 'tasocitinib':ti,ab

#11 'filgotinib'/exp OR 'filgotinib' OR 'GLPG0634':ti,ab

#12 'upadacitinib'/exp OR 'upadacitinib' OR 'ABT-494':ti,ab

#13 'randomized controlled trial'/exp OR 'controlled clinical trial'/exp OR randomized:ti,ab OR placebo:ti,ab OR 'drug therapy':lnk OR randomly:ti,ab OR trial:ti,ab OR groups:ti,ab

#14 'clinical article'/exp OR 'controlled study'/exp OR 'major clinical study'/exp OR 'prospective study'/exp OR 'cohort analysis'/exp OR 'cohort':ti,ab OR 'compared':ti,ab OR 'groups':ti,ab OR 'case control':ti,ab OR 'multivariate':ti,ab

#15 #2 OR #3 OR #4 OR #5 OR #6 OR #7 OR #8 OR #9 OR #10 OR #11 OR #12

#16 #1 AND #13 AND #15

#17 #16 NOT #14NOT ("cohort studies"[mesh] OR "case-control studies"[mesh] OR "comparative study"[pt] OR "risk factors"[mesh] OR "cohort"[tw] OR "compared"[tw] OR "groups"[tw] OR "case control"[tw] OR "multivariate"[tw])

# Supplementary Figures and Tables


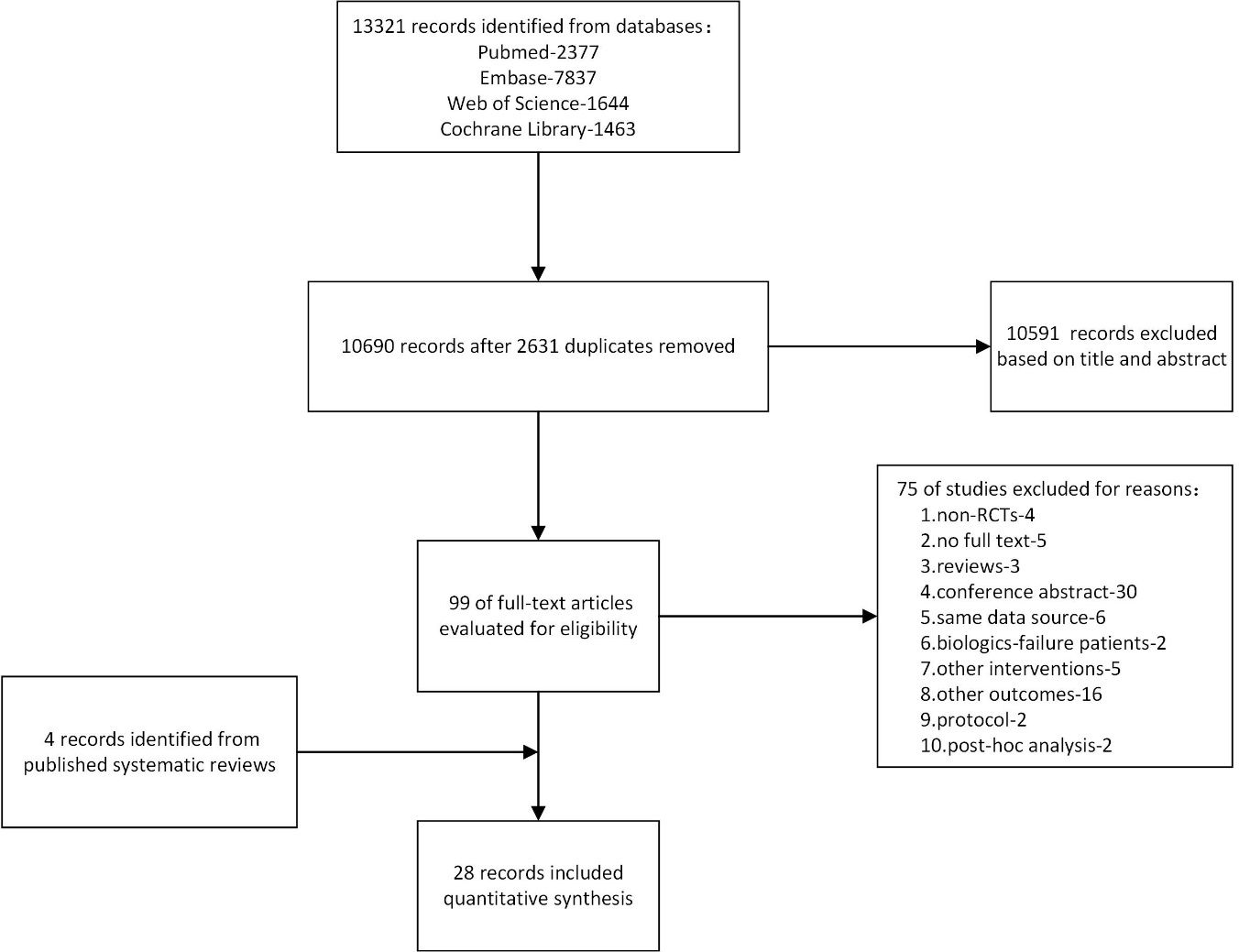


**Supplementary Figure S1** Flow diagram of literature screening

**
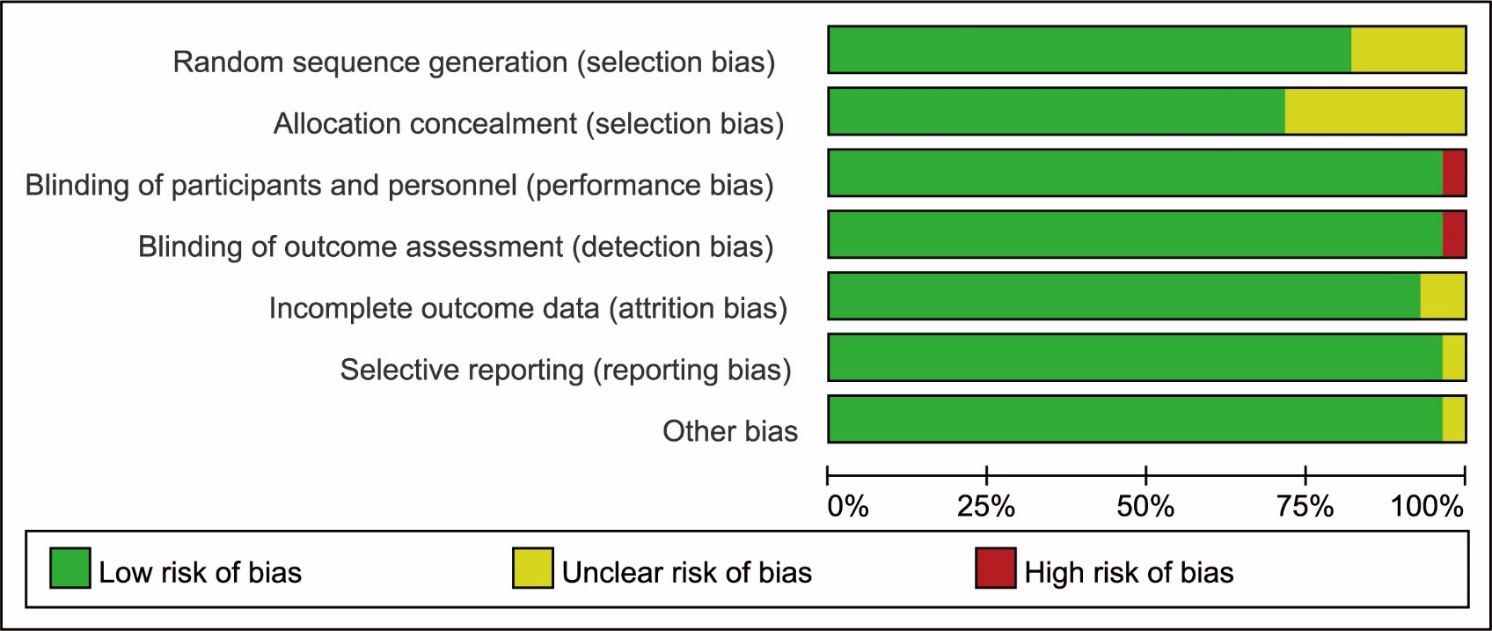
**

**Supplementary Figure S2** Risk of bias summary: review authors' judgements about each risk of bias item for each included study


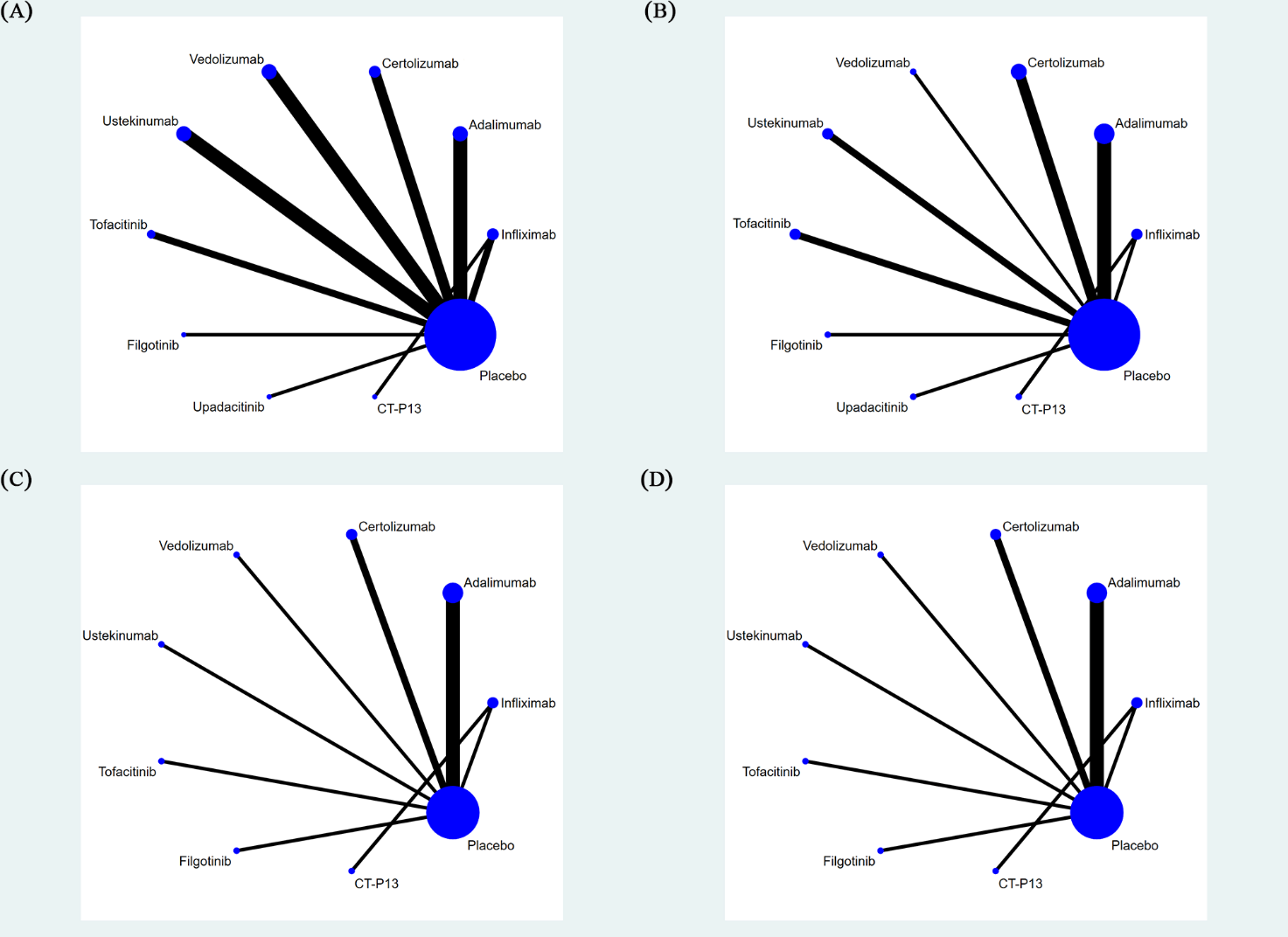


**Supplementary Figure S3** Network plot for safety. (A) AEs in induction phase. (B) infections or serious/severe infections in induction phase. (C) AEs in maintenance phase. (C) infections or serious/severe infections in maintenance phase.


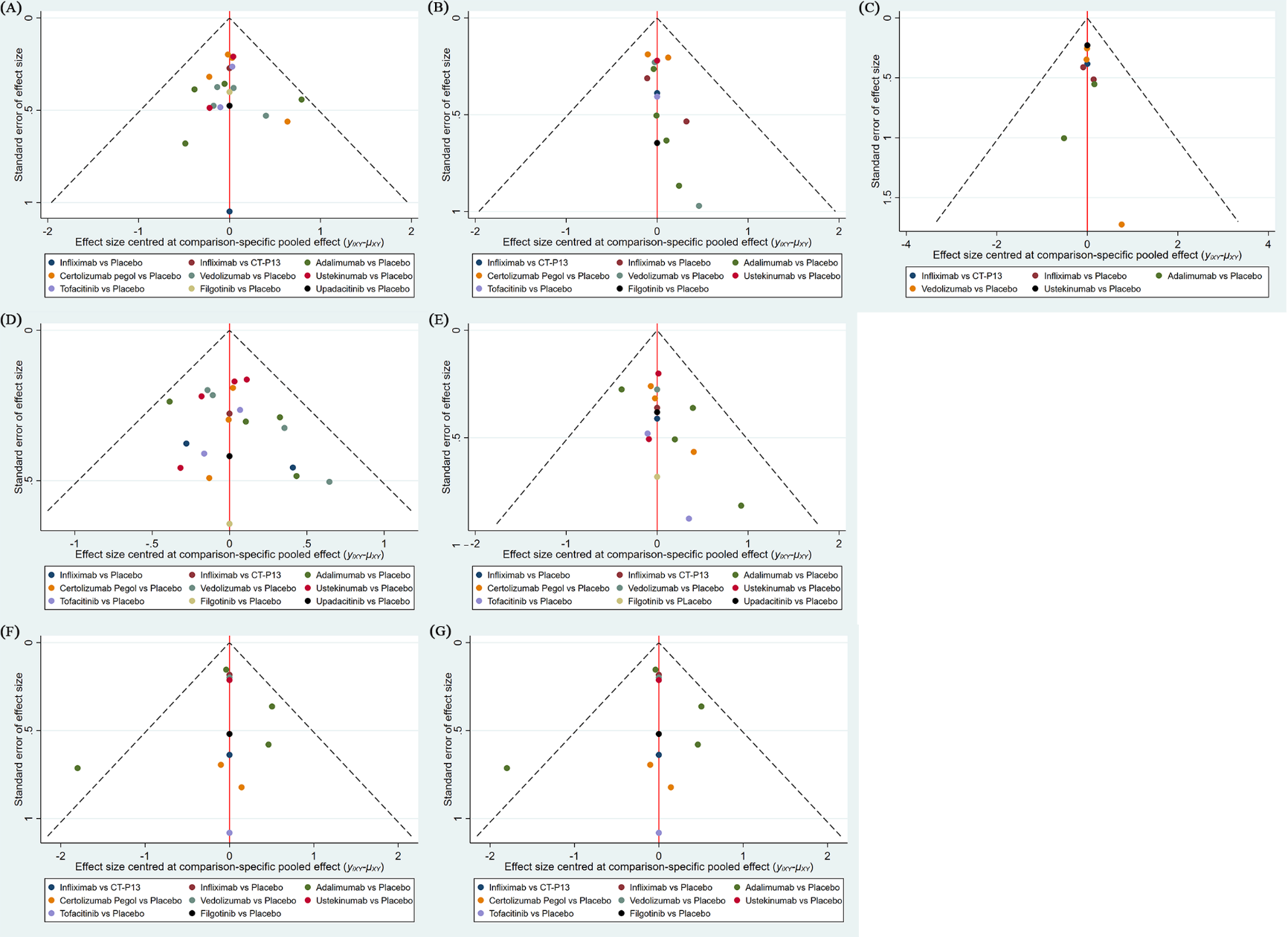


**Supplementary Figure S4** Funnel plot for: (A) induction of remission. (B) maintenance of remission. (C) steroids-free remission. (D) AEs in induction phase. (E) infections or serious/severe infections in induction phase. (F) AEs in maintenance phase. (G) infections or serious/severe infections in maintenance phase.

**Supplementary Table S1** Consistency and inconsistency model

| **Outcomes for assessment** | **Consistency model** | **Inconsistency model** | |
| --- | --- | --- | --- |
|  | Random Effects Standard Deviation, Median (95% CI) | Random Effects Standard Deviation, Median (95% CI) | Inconsistency Standard Deviation, Median (95% CI) |
| Induction of remission | 0.13 (0.01, 0.48) | 0.13 (0.01, 0.50) | 1.02 (0.05, 1.99) |
| Maintenance of remission | 0.14 (0.01, 0.59) | 0.14 (0.01, 0.59) | 0.82 (0.04, 1.60) |
| Steroids-free remission | 0.27 (0.01, 1.32) | 0.28 (0.02, 1.33) | 0.81 (0.04, 1.57) |
| AEs in induction phase | 0.18 (0.02, 0.52) | 0.18 (0.02, 0.52) | 0.37 (0.02, 0.71) |
| Infections or serious/severe infections in induction phase | 0.24 (0.03, 0.65) | 0.24 (0.02, 0.66) | 0.36 (0.02, 0.71) |
| AEs in maintenance phase | 0.80 (0.04, 2.18) | 0.84 (0.10, 2.19) | 1.17 (0.06, 2.31) |
| Infections or serious/severe infections in maintenance phase | 0.72 (0.09, 1.29) | 0.71 (0.08, 1.28) | 0.66 (0.03, 1.30) |

| **Supplementary Table S2** Pairwise comparisons of AEs and infections or serious/severe infections in induction phase | | | | | | | | | |
| --- | --- | --- | --- | --- | --- | --- | --- | --- | --- |
| Intervention  Comparator | Odds ratios (95% confidence interval) | | | | | | | | |
|  | Infliximab | Adalimumab | Certolizumab | Vedolizumab | Ustekinumab | Tofacitinib | Filgotinib | Upadacitinib | CT-P13 |
| **AEs** | | | | | | | | | |
| Adalimumab | 1.85 (0.84, 4.00) | - |  |  |  |  |  |  |  |
| Certolizumab | 1.19 (0.53, 2.74) | 0.64 (0.37, 1.19) | - |  |  |  |  |  |  |
| Vedolizumab | 1.22 (0.53, 2.86) | 0.66 (0.36, 1.20) | 1.02 (0.52, 1.87) | - |  |  |  |  |  |
| Ustekinumab | 1.59 (0.70, 3.82) | 0.86 (0.46, 1.71) | 1.34 (0.69, 2.67) | 1.30 (0.69, 2.77) | - |  |  |  |  |
| Tofacitinib | 1.57 (0.65, 3.91) | 0.85 (0.43, 1.74) | 1.31 (0.65, 2.74) | 1.29 (0.64, 2.78) | 0.98 (0.45, 2.08) | - |  |  |  |
| Filgotinib | 0.70 (0.14, 3.23) | 0.38 (0.09, 1.63) | 0.59 (0.13, 2.50) | 0.57 (0.13, 2.56) | 0.44 (0.09, 1.90) | 0.45 (0.09, 1.96) | - |  |  |
| Upadacitinib | 0.80 (0.25, 2.59) | 0.43 (0.16, 1.25) | 0.66 (0.24, 1.94) | 0.65 (0.23, 1.98) | 0.49 (0.17, 1.45) | 0.50 (0.17, 1.55) | 1.14 (0.22, 6.36) | - |  |
| CT-P13 | 1.38 (0.67, 2.87) | 0.75 (0.26, 2.19) | 1.16 (0.39, 3.40) | 1.13 (0.39, 3.51) | 0.87 (0.28, 2.61) | 0.88 (0.28, 2.80) | 1.97 (0.37, 11.15) | 1.73 (0.43, 6.75) | - |
| Placebo | 1.38 (0.71, 2.84) | 0.75 (0.52, 1.13) | 1.16 (0.75, 1.78) | 1.14 (0.74, 1.86) | 0.87 (0.51, 1.42) | 0.88 (0.50, 1.55) | 1.97 (0.50, 8.32) | 1.76 (0.65, 4.45) | 1.00 (0.37, 2.68) |
| **Infections or serious/severe infections** | | | | | | | | | |
| Adalimumab | 1.47 (0.44, 4.46) | - |  |  |  |  |  |  |  |
| Certolizumab | 0.93 (0.28, 2.91) | 0.63 (0.30, 1.38) | - |  |  |  |  |  |  |
| Vedolizumab | 1.58 (0.41, 5.83) | 1.05 (0.42, 3.02) | 1.68 (0.64, 4.69) | - |  |  |  |  |  |
| Ustekinumab | 1.79 (0.53, 6.19) | 1.19 (0.56, 3.00) | 1.92 (0.85, 4.66) | 1.15 (0.38, 3.34) | - |  |  |  |  |
| Tofacitinib | 1.70 (0.35, 7.86) | 1.16 (0.33, 4.19) | 1.84 (0.50, 6.55) | 1.09 (0.26, 4.55) | 0.96 (0.25, 3.55) | - |  |  |  |
| Filgotinib | 0.79 (0.12, 5.25) | 0.53 (0.11, 2.61) | 0.84 (0.17, 4.63) | 0.50 (0.08, 2.81) | 0.44 (0.08, 2.33) | 0.47 (0.07, 3.08) | - |  |  |
| Upadacitinib | 0.53 (0.13, 2.23) | 0.37 (0.12, 1.17) | 0.58 (0.19, 1.77) | 0.34 (0.09, 1.27) | **0.30 (0.09, 0.95)** | 0.32 (0.07, 1.45) | 0.68 (0.11, 4.11) | - |  |
| CT-P13 | 0.97 (0.37, 2.50) | 0.65 (0.15, 3.12) | 1.01 (0.23, 4.81) | 0.61 (0.12, 3.20) | 0.54 (0.11, 2.58) | 0.56 (0.10, 3.58) | 1.21 (0.14, 9.95) | 1.79 (0.33, 10.18) | - |
| Placebo | 1.16 (0.41, 3.20) | 0.78 (0.49, 1.39) | 1.25 (0.73, 2.20) | 0.74 (0.31, 1.74) | 0.65 (0.34, 1.23) | 0.68 (0.22, 2.21) | 1.49 (0.31, 6.83) | 2.16 (0.82, 6.07) | 1.22 (0.29, 4.87) |

Value of OR <1 favors the intervention over the comparator.

| **Supplementary Table S3** Rank probability for AEs and infections or serious/severe infections in induction phase | | | | | | | | | | |
| --- | --- | --- | --- | --- | --- | --- | --- | --- | --- | --- |
| **Drug** | **Rank 1** | **Rank 2** | **Rank 3** | **Rank 4** | **Rank 5** | **Rank 6** | **Rank 7** | **Rank 8** | **Rank 9** | **Rank 10** |
| **AEs** | | | | | | | | | | |
| Infliximab | 0.1 | 0.23 | 0.25 | 0.15 | 0.09 | 0.06 | 0.05 | 0.04 | 0.02 | 0.01 |
| Adalimumab | 0 | 0 | 0.01 | 0.01 | 0.03 | 0.04 | 0.09 | 0.17 | 0.29 | 0.36 |
| Certolizumab | 0.02 | 0.09 | 0.18 | 0.2 | 0.18 | 0.13 | 0.09 | 0.06 | 0.04 | 0.01 |
| Vedolizumab | 0.02 | 0.09 | 0.16 | 0.18 | 0.18 | 0.13 | 0.09 | 0.08 | 0.04 | 0.02 |
| Ustekinumab | 0 | 0.01 | 0.03 | 0.05 | 0.08 | 0.1 | 0.14 | 0.21 | 0.21 | 0.16 |
| Tofacitinib | 0.01 | 0.02 | 0.05 | 0.07 | 0.09 | 0.1 | 0.13 | 0.17 | 0.19 | 0.17 |
| Filgotinib | 0.48 | 0.18 | 0.08 | 0.06 | 0.04 | 0.03 | 0.03 | 0.03 | 0.03 | 0.05 |
| Upadacitinib | 0.32 | 0.29 | 0.12 | 0.08 | 0.05 | 0.04 | 0.03 | 0.03 | 0.03 | 0.02 |
| CT-P13 | 0.04 | 0.08 | 0.11 | 0.13 | 0.08 | 0.07 | 0.08 | 0.1 | 0.12 | 0.2 |
| Placebo | 0 | 0 | 0.02 | 0.07 | 0.19 | 0.3 | 0.28 | 0.12 | 0.02 | 0 |
| **infections or serious/severe infections** | | | | | | | | | | |
| Infliximab | 0.04 | 0.15 | 0.18 | 0.16 | 0.13 | 0.1 | 0.08 | 0.07 | 0.06 | 0.03 |
| Adalimumab | 0 | 0.01 | 0.03 | 0.06 | 0.09 | 0.13 | 0.2 | 0.23 | 0.17 | 0.07 |
| Certolizumab | 0.03 | 0.17 | 0.24 | 0.2 | 0.17 | 0.09 | 0.05 | 0.03 | 0.01 | 0 |
| Vedolizumab | 0.01 | 0.03 | 0.05 | 0.06 | 0.08 | 0.1 | 0.15 | 0.17 | 0.2 | 0.17 |
| Ustekinumab | 0 | 0.01 | 0.01 | 0.02 | 0.04 | 0.07 | 0.14 | 0.19 | 0.27 | 0.25 |
| Tofacitinib | 0.02 | 0.04 | 0.06 | 0.06 | 0.06 | 0.07 | 0.1 | 0.12 | 0.15 | 0.31 |
| Filgotinib | 0.27 | 0.19 | 0.11 | 0.08 | 0.05 | 0.05 | 0.05 | 0.06 | 0.06 | 0.08 |
| Upadacitinib | 0.47 | 0.25 | 0.12 | 0.07 | 0.04 | 0.02 | 0.01 | 0.01 | 0.01 | 0 |
| CT-P13 | 0.15 | 0.16 | 0.15 | 0.1 | 0.07 | 0.07 | 0.07 | 0.07 | 0.07 | 0.09 |
| Placebo | 0 | 0.01 | 0.06 | 0.17 | 0.27 | 0.3 | 0.14 | 0.04 | 0.01 | 0 |

| **Supplementary Table S4** Pairwise comparisons of AEs and infections or serious/severe infections in maintenance phase | | | | | | | | |
| --- | --- | --- | --- | --- | --- | --- | --- | --- |
| Intervention  Comparator | Odds ratios (95% confidence interval) | | | | | | | |
|  | Infliximab | Adalimumab | Certolizumab | Vedolizumab | Ustekinumab | Tofacitinib | Filgotinib | CT-P13 |
| **AEs** | | | | | | | | |
| Adalimumab | 0.41 (0.01, 18.43) | - |  |  |  |  |  |  |
| Certolizumab | 0.39 (0.00, 17.32) | 1.03 (0.09, 6.72) | - |  |  |  |  |  |
| Vedolizumab | 0.40 (0.00, 21.23) | 0.99 (0.09, 8.97) | 0.97 (0.09, 14.03) | - |  |  |  |  |
| Ustekinumab | 0.47 (0.00, 29.10) | 1.24 (0.06, 14.60) | 1.22 (0.06, 21.92) | 1.26 (0.05, 22.24) | - |  |  |  |
| Tofacitinib | 0.26 (0.00, 18.76) | 0.70 (0.03, 8.83) | 0.69 (0.04, 13.66) | 0.70 (0.03, 13.18) | 0.56 (0.02, 17.93) | - |  |  |
| Filgotinib | 0.48 (0.00, 36.94) | 1.20 (0.05, 18.67) | 1.22 (0.05, 28.27) | 1.23 (0.04, 28.03) | 0.99 (0.03, 35.71) | 1.76 (0.05, 63.12) | - |  |
| CT-P13 | 0.73 (0.06, 8.84) | 1.84 (0.02, 228.23) | 1.88 (0.02, 290.53) | 1.86 (0.02, 304.34) | 1.56 (0.01, 319.73) | 2.82 (0.02, 559.64) | 1.55 (0.01, 338.87) | - |
| Placebo | 0.40 (0.01, 12.35) | 1.02 (0.20, 3.04) | 1.01 (0.19, 5.60) | 1.03 (0.14, 5.79) | 0.83 (0.08, 9.25) | 1.47 (0.12, 16.55) | 0.84 (0.06, 10.65) | 0.53 (0.00, 34.70) |
| **infections or serious/severe infections** | | | | | | | | |
| Adalimumab | 0.54 (0.09, 4.07) | - |  |  |  |  |  |  |
| Certolizumab | 0.26 (0.02, 2.48) | 0.47 (0.07, 2.58) | - |  |  |  |  |  |
| Vedolizumab | 0.58 (0.05, 6.49) | 1.08 (0.14, 6.75) | 2.27 (0.22, 25.12) | - |  |  |  |  |
| Ustekinumab | 0.75 (0.07, 7.81) | 1.37 (0.18, 8.70) | 2.90 (0.31, 29.49) | 1.28 (0.11, 14.35) | - |  |  |  |
| Tofacitinib | 0.15 (0.00, 3.13) | 0.27 (0.01, 3.76) | 0.57 (0.02, 11.48) | 0.25 (0.01, 5.35) | 0.20 (0.01, 4.31) | - |  |  |
| Filgotinib | 0.46 (0.03, 6.00) | 0.84 (0.09, 6.65) | 1.78 (0.15, 22.71) | 0.79 (0.06, 10.91) | 0.61 (0.05, 7.83) | 3.14 (0.13, 130.87) | - |  |
| CT-P13 | 1.34 (0.17, 10.65) | 2.41 (0.14, 39.67) | 5.39 (0.25, 118.30) | 2.29 (0.10, 55.64) | 1.80 (0.08, 44.72) | 9.24 (0.22, 675.90) | 2.94 (0.11, 92.99) | - |
| Placebo | 0.72 (0.13, 3.94) | 1.32 (0.49, 3.11) | 2.81 (0.61, 14.37) | 1.24 (0.22, 7.04) | 0.96 (0.18, 5.34) | 4.80 (0.39, 134.03) | 1.56 (0.23, 11.07) | 0.53 (0.04, 7.59) |

Value of OR <1 favors the intervention over the comparator.

| **Supplementary Table S5** Rank probability for AEs and infections or serious/severe infections in maintenance phase | | | | | | | | | |
| --- | --- | --- | --- | --- | --- | --- | --- | --- | --- |
| **Drug** | **Rank 1** | **Rank 2** | **Rank 3** | **Rank 4** | **Rank 5** | **Rank 6** | **Rank 7** | **Rank 8** | **Rank 9** |
| **AEs** | | | | | | | | | |
| Infliximab | 0.04 | 0.1 | 0.06 | 0.05 | 0.04 | 0.05 | 0.09 | 0.24 | 0.31 |
| Adalimumab | 0.06 | 0.13 | 0.15 | 0.15 | 0.15 | 0.14 | 0.11 | 0.07 | 0.04 |
| Certolizumab | 0.08 | 0.12 | 0.14 | 0.14 | 0.15 | 0.14 | 0.12 | 0.07 | 0.04 |
| Vedolizumab | 0.11 | 0.13 | 0.14 | 0.12 | 0.12 | 0.12 | 0.12 | 0.08 | 0.06 |
| Ustekinumab | 0.09 | 0.1 | 0.1 | 0.09 | 0.09 | 0.13 | 0.17 | 0.11 | 0.13 |
| Tofacitinib | 0.3 | 0.17 | 0.12 | 0.09 | 0.08 | 0.08 | 0.07 | 0.05 | 0.05 |
| Filgotinib | 0.14 | 0.11 | 0.09 | 0.08 | 0.08 | 0.1 | 0.15 | 0.1 | 0.16 |
| CT-P13 | 0.17 | 0.09 | 0.06 | 0.04 | 0.04 | 0.05 | 0.09 | 0.25 | 0.21 |
| Placebo | 0.01 | 0.05 | 0.15 | 0.25 | 0.25 | 0.18 | 0.08 | 0.03 | 0 |
| **infections or serious/severe infections** | | | | | | | | | |
| Infliximab | 0.01 | 0.03 | 0.05 | 0.07 | 0.08 | 0.1 | 0.17 | 0.34 | 0.16 |
| Adalimumab | 0.01 | 0.07 | 0.18 | 0.24 | 0.19 | 0.13 | 0.1 | 0.06 | 0.02 |
| Certolizumab | 0.25 | 0.33 | 0.18 | 0.1 | 0.06 | 0.04 | 0.03 | 0.02 | 0.01 |
| Vedolizumab | 0.05 | 0.1 | 0.15 | 0.15 | 0.14 | 0.12 | 0.12 | 0.09 | 0.08 |
| Ustekinumab | 0.02 | 0.06 | 0.1 | 0.11 | 0.12 | 0.12 | 0.17 | 0.15 | 0.14 |
| Tofacitinib | 0.53 | 0.17 | 0.08 | 0.05 | 0.04 | 0.03 | 0.03 | 0.03 | 0.03 |
| Filgotinib | 0.09 | 0.19 | 0.18 | 0.12 | 0.1 | 0.08 | 0.09 | 0.08 | 0.07 |
| CT-P13 | 0.03 | 0.05 | 0.06 | 0.06 | 0.05 | 0.06 | 0.08 | 0.15 | 0.46 |
| Placebo | 0 | 0 | 0.02 | 0.09 | 0.23 | 0.32 | 0.22 | 0.09 | 0.02 |
