# Supplementary material for: Systematic Review and Network Meta-Analysis: Comparative Efficacy and Safety of Biosimilars, Biologics and JAK1 Inhibitors for Active Crohn Disease
Source: Front Pharmacol. 2021 Apr 14;12:655865. doi: 10.3389/fphar.2021.655865 (PMC8080031; doi:10.3389/fphar.2021.655865)
Supplement: Supplementary file 2 [file datasheet2.docx]

model {

for (i in 1:ns) {

# Likelihood for each arm

for (k in 1:na[i]) {

r[i, k] ~ dbin(p[i, k], n[i, k])

logit(p[i, k]) <- mu[i] + delta[i, k]

}

# Study-level relative effects

# The arms are given in the order (arm_1, arm_2, ..., arm_{n_a-1}, arm_{n_a}).

# The relative effects are parameterized as d[arm_1, arm_k].

w[i, 1] <- 0

delta[i, 1] <- 0

for (k in 2:na[i]) { # parameterize multi-arm trials using a trick to avoid dmnorm

delta[i, k] ~ dnorm(md[i, k], taud[i, k])

md[i, k] <- d[t[i, 1], t[i, k]] + sw[i, k]

taud[i, k] <- tau.d * 2 * (k - 1) / k

w[i, k] <- delta[i, k] - d[t[i, 1], t[i, k]]

sw[i, k] <- sum(w[i, 1:k-1]) / (k - 1)

}

}

# Relative effect matrix

d[1,1] <- 0

d[1,2] <- d.A.J + d.J.B

d[1,3] <- d.A.J + d.J.C

d[1,4] <- d.A.J + d.J.D

d[1,5] <- d.A.J + d.J.E

d[1,6] <- d.A.J + d.J.F

d[1,7] <- d.A.J + d.J.G

d[1,8] <- d.A.J + d.J.H

d[1,9] <- d.A.I

d[1,10] <- d.A.J

d[2,1] <- -d.A.J + -d.J.B

d[2,2] <- 0

d[2,3] <- -d.J.B + d.J.C

d[2,4] <- -d.J.B + d.J.D

d[2,5] <- -d.J.B + d.J.E

d[2,6] <- -d.J.B + d.J.F

d[2,7] <- -d.J.B + d.J.G

d[2,8] <- -d.J.B + d.J.H

d[2,9] <- d.A.I + -d.A.J + -d.J.B

d[2,10] <- -d.J.B

d[3,1] <- -d.A.J + -d.J.C

d[3,2] <- d.J.B + -d.J.C

d[3,3] <- 0

d[3,4] <- -d.J.C + d.J.D

d[3,5] <- -d.J.C + d.J.E

d[3,6] <- -d.J.C + d.J.F

d[3,7] <- -d.J.C + d.J.G

d[3,8] <- -d.J.C + d.J.H

d[3,9] <- d.A.I + -d.A.J + -d.J.C

d[3,10] <- -d.J.C

d[4,1] <- -d.A.J + -d.J.D

d[4,2] <- d.J.B + -d.J.D

d[4,3] <- d.J.C + -d.J.D

d[4,4] <- 0

d[4,5] <- -d.J.D + d.J.E

d[4,6] <- -d.J.D + d.J.F

d[4,7] <- -d.J.D + d.J.G

d[4,8] <- -d.J.D + d.J.H

d[4,9] <- d.A.I + -d.A.J + -d.J.D

d[4,10] <- -d.J.D

d[5,1] <- -d.A.J + -d.J.E

d[5,2] <- d.J.B + -d.J.E

d[5,3] <- d.J.C + -d.J.E

d[5,4] <- d.J.D + -d.J.E

d[5,5] <- 0

d[5,6] <- -d.J.E + d.J.F

d[5,7] <- -d.J.E + d.J.G

d[5,8] <- -d.J.E + d.J.H

d[5,9] <- d.A.I + -d.A.J + -d.J.E

d[5,10] <- -d.J.E

d[6,1] <- -d.A.J + -d.J.F

d[6,2] <- d.J.B + -d.J.F

d[6,3] <- d.J.C + -d.J.F

d[6,4] <- d.J.D + -d.J.F

d[6,5] <- d.J.E + -d.J.F

d[6,6] <- 0

d[6,7] <- -d.J.F + d.J.G

d[6,8] <- -d.J.F + d.J.H

d[6,9] <- d.A.I + -d.A.J + -d.J.F

d[6,10] <- -d.J.F

d[7,1] <- -d.A.J + -d.J.G

d[7,2] <- d.J.B + -d.J.G

d[7,3] <- d.J.C + -d.J.G

d[7,4] <- d.J.D + -d.J.G

d[7,5] <- d.J.E + -d.J.G

d[7,6] <- d.J.F + -d.J.G

d[7,7] <- 0

d[7,8] <- -d.J.G + d.J.H

d[7,9] <- d.A.I + -d.A.J + -d.J.G

d[7,10] <- -d.J.G

d[8,1] <- -d.A.J + -d.J.H

d[8,2] <- d.J.B + -d.J.H

d[8,3] <- d.J.C + -d.J.H

d[8,4] <- d.J.D + -d.J.H

d[8,5] <- d.J.E + -d.J.H

d[8,6] <- d.J.F + -d.J.H

d[8,7] <- d.J.G + -d.J.H

d[8,8] <- 0

d[8,9] <- d.A.I + -d.A.J + -d.J.H

d[8,10] <- -d.J.H

d[9,1] <- -d.A.I

d[9,2] <- -d.A.I + d.A.J + d.J.B

d[9,3] <- -d.A.I + d.A.J + d.J.C

d[9,4] <- -d.A.I + d.A.J + d.J.D

d[9,5] <- -d.A.I + d.A.J + d.J.E

d[9,6] <- -d.A.I + d.A.J + d.J.F

d[9,7] <- -d.A.I + d.A.J + d.J.G

d[9,8] <- -d.A.I + d.A.J + d.J.H

d[9,9] <- 0

d[9,10] <- -d.A.I + d.A.J

d[10,1] <- -d.A.J

d[10,2] <- d.J.B

d[10,3] <- d.J.C

d[10,4] <- d.J.D

d[10,5] <- d.J.E

d[10,6] <- d.J.F

d[10,7] <- d.J.G

d[10,8] <- d.J.H

d[10,9] <- d.A.I + -d.A.J

d[10,10] <- 0

# Study baseline priors

for (i in 1:ns) {

mu[i] ~ dnorm(0, 1.069E-3)

}

# Variance prior

sd.d ~ dunif(0, 2.039E0)

tau.d <- pow(sd.d, -2)

# Effect parameter priors

d.A.I ~ dnorm(0, 1.069E-3)

d.A.J ~ dnorm(0, 1.069E-3)

d.J.B ~ dnorm(0, 1.069E-3)

d.J.C ~ dnorm(0, 1.069E-3)

d.J.D ~ dnorm(0, 1.069E-3)

d.J.E ~ dnorm(0, 1.069E-3)

d.J.F ~ dnorm(0, 1.069E-3)

d.J.G ~ dnorm(0, 1.069E-3)

d.J.H ~ dnorm(0, 1.069E-3)

}

list(

ns = 21,

na = c(2, 2, 2, 2, 2, 2, 2, 2, 2, 2, 2, 2, 2, 2, 2, 2, 2, 2, 2, 2, 2),

t = structure(.Data = c(10, 8, 10, 2, 10, 2, 10, 7, 10, 4, 10, 2, 10, 4, 10, 4, 10, 3, 10, 6, 10, 1, 10, 5, 10, 3, 10, 6, 10, 3, 10, 1, 10, 5, 10, 2, 10, 4, 10, 3, 1, 9), .Dim = c(21, 2)),

r = structure(.Data = c(6, 51, 9, 58, 7, 38, 10, 60, 12, 43, 12, 34, 10, 32, 6, 16, 57, 71, 33, 81, 58, 101, 9, 13, 53, 68, 7, 24, 17, 53, 1, 27, 37, 133, 3, 17, 8, 14, 5, 25, 49, 47), .Dim = c(21, 2)),

n = structure(.Data = c(37, 183, 74, 225, 103, 102, 44, 128, 58, 127, 166, 159, 148, 220, 50, 51, 326, 329, 90, 187, 170, 169, 53, 51, 209, 215, 34, 104, 73, 218, 24, 83, 209, 418, 23, 67, 78, 79, 25, 65, 109, 111), .Dim = c(21, 2))

)

list(

d.A.I = -0.017424727876257692,

d.A.J = -1.2385515833513199,

d.J.B = 0.5221388584737086,

d.J.C = 0.46575044376183006,

d.J.D = 0.4087440712512769,

d.J.E = 0.19742627429648674,

d.J.F = 1.294859580062647,

d.J.G = 0.5121706941113281,

d.J.H = -0.933158064807369,

mu = c(0.46584926807391747, -3.227438678354477, -2.3085026045738917, -1.9006344093627328, -2.258729934338934, -2.161151573062724, -3.915508663766971, -0.8901784809392321, -1.0737312939809802, -0.6600596353909906, -0.39770138336668404, -1.3747234159475892, -0.6562445919285572, -0.62253991453354, -0.7124088414649381, -2.297329004190748, -2.4547188995122844, -1.577919775671228, -2.5361363293157444, -1.8057838239936213, 0.31064358231726197),

delta = structure(.Data = c(NA, 2.677091141009346, NA, 1.6318152948007691, NA, 3.6537540392196917, NA, 2.4915987959585646, NA, 2.304155439080472, NA, -0.2427895439884522, NA, -0.9792840838175554, NA, 1.1343871066114335, NA, -0.22088563287612267, NA, 0.34760265175761773, NA, 0.5216436677295031, NA, 1.5304991604000475, NA, 0.08170721343582005, NA, -0.259835646452456, NA, -0.590428444969947, NA, 2.97004585169234, NA, 0.2861535009376481, NA, -1.2967295531077612, NA, 1.4177710202541594, NA, 1.155337442299241, NA, -0.4673256549673949), .Dim = c(21, 2)),

sd.d = 0.1076998191898522

)

list(

d.A.I = 0.31552100528319643,

d.A.J = -1.8881416632288146,

d.J.B = 1.390857131389974,

d.J.C = 0.00975414842635064,

d.J.D = 0.18618761601569067,

d.J.E = 0.3671393208628963,

d.J.F = 0.2308408267222097,

d.J.G = 0.8739248359266317,

d.J.H = 1.6000873025114262,

mu = c(-2.172512995868294, -2.7044104353746254, -1.6217066111651743, -0.8604696270624115, -1.9117449744005064, -1.1561911875218172, -1.8050693010108783, -0.8332246846705478, -0.9314567052688169, 0.6478790202565221, -0.7364957009564543, -2.123293784195346, -0.3352034077360714, 0.22535385724713097, -1.0694126771121322, -5.200150819410262, -2.221801701295823, -2.4334863307145507, -0.8024474224517497, -0.42071956166702384, -1.3084063184206847),

delta = structure(.Data = c(NA, -1.6809844905110651, NA, 0.1770170326067546, NA, 1.9363295669256428, NA, 0.3970413472187826, NA, 0.1232617289630542, NA, 1.9703746008322698, NA, -0.5619905126906637, NA, -0.6576715548015089, NA, 0.3508684832919442, NA, -0.27675948826595964, NA, 0.052725459678627185, NA, -0.09624939547277345, NA, 1.0859343973807905, NA, 2.1832451110469373, NA, -0.27383362266246947, NA, 1.063744749515911, NA, 1.3500654033195096, NA, 4.5134728824643995, NA, 1.108908258411568, NA, 0.5456981219920904, NA, 0.15410089944150696), .Dim = c(21, 2)),

sd.d = 0.2996297658742619

)

list(

d.A.I = 1.0721500208475483,

d.A.J = -0.8147480621282386,

d.J.B = 1.2921090039181669,

d.J.C = 0.6483103881999828,

d.J.D = 0.26981553329909147,

d.J.E = 0.5005823324501292,

d.J.F = 0.6558719665636763,

d.J.G = -0.7361176445815878,

d.J.H = -1.4259334510656436,

mu = c(-0.3091264876056823, -2.600509765388175, -1.206540001999719, -2.0712478411469193, -1.8736557822409103, -2.812573749637949, -2.9841631362996655, -1.1075802214386659, -2.104629861983045, -0.48818971118208027, -0.6941361334258033, -0.2947825753058304, -1.0942566612850737, -0.22240035276561088, -0.26160313881148434, -4.913050055999482, -1.1895384643313511, -3.038772701788665, -1.5575378265377289, -2.0897138090030616, -0.30588924400933143),

delta = structure(.Data = c(NA, 0.18316108438956175, NA, 1.1132591361149267, NA, 1.4183625553006656, NA, 1.1612420874434968, NA, 0.9769263476932267, NA, 2.910158357194305, NA, 0.4104654889807273, NA, -0.8316662980118328, NA, 0.6000687606589516, NA, 0.5952479758848335, NA, 0.6686320973057418, NA, 0.6676320299328522, NA, 1.0019077900754298, NA, -1.9905204061516082, NA, -1.0508193072887964, NA, 0.9707795754848023, NA, 0.24552392123645916, NA, 0.9664445843057261, NA, 1.4450867071384335, NA, -0.04831843725328444, NA, 0.17215379797877933), .Dim = c(21, 2)),

sd.d = 0.7879779958232689

)

list(

d.A.I = -0.4391610220159927,

d.A.J = 0.014307257894882408,

d.J.B = 1.530913850928696,

d.J.C = 0.7275970813979367,

d.J.D = 0.4618497776604784,

d.J.E = 1.562114575079132,

d.J.F = -0.4081171425877779,

d.J.G = -0.3396208920176831,

d.J.H = 0.06379120043034836,

mu = c(-1.4732850988178647, -2.0725634420097085, -1.397245051830049, -2.1623390978034305, -1.6091889155884975, -2.1393343662236095, -2.2237962829221485, -1.316916838040095, -1.539239299635114, 0.09130048949896985, -0.30703508575163013, -1.0926055779032173, -1.085303873549354, -3.180939007919801, -1.3813640816744213, -4.5191545139979175, -1.2462842752100616, -2.545131892917997, -2.05414328842007, -0.16242252942314028, 0.31997768954414696),

delta = structure(.Data = c(NA, -1.6409236156251459, NA, 0.24810794469282693, NA, 3.3200825081224847, NA, 2.500874397322203, NA, 0.9648979519898901, NA, 1.868896773126428, NA, 0.8991676595605501, NA, 3.1130868856702083, NA, -0.12316791935979743, NA, -0.8179173891624667, NA, 1.4820638938684954, NA, 0.931514971906569, NA, 0.5909353300940813, NA, 0.36026384923398813, NA, -1.7280143725384742, NA, -1.2479600542051288, NA, 1.0140388660156907, NA, -0.734429966129124, NA, 1.6956770660011276, NA, 2.83605401550153, NA, 0.4967155309428761), .Dim = c(21, 2)),

sd.d = 1.6127004594001944

)

modelCheck('Consistency Model.cons.model')

modelData('Consistency Model.cons.data')

modelCompile(4)

modelInits('Consistency Model.cons.inits1', 1)

modelInits('Consistency Model.cons.inits2', 2)

modelInits('Consistency Model.cons.inits3', 3)

modelInits('Consistency Model.cons.inits4', 4)

modelSetAP('slice', 20000)

modelUpdate(20000)

samplesSet('d.A.I')

samplesSet('d.A.J')

samplesSet('d.J.B')

samplesSet('d.J.C')

samplesSet('d.J.D')

samplesSet('d.J.E')

samplesSet('d.J.F')

samplesSet('d.J.G')

samplesSet('d.J.H')

samplesSet('sd.d')

modelUpdate(200000)

samplesCoda('*', 'Consistency Model.cons')
