# Supplementary material for: Systematic Review and Network Meta-Analysis: Comparative Efficacy and Safety of Biosimilars, Biologics and JAK1 Inhibitors for Active Crohn Disease
Source: Front Pharmacol. 2021 Apr 14;12:655865. doi: 10.3389/fphar.2021.655865 (PMC8080031; doi:10.3389/fphar.2021.655865)
Supplement: Supplementary file 3 [file datasheet3.docx]

model {

for (i in 1:ns) {

# Likelihood for each arm

for (k in 1:na[i]) {

r[i, k] ~ dbin(p[i, k], n[i, k])

logit(p[i, k]) <- mu[i] + delta[i, k]

}

# Study-level relative effects

# The arms are given in the order (arm_1, arm_2, ..., arm_{n_a-1}, arm_{n_a}).

# The relative effects are parameterized as d[arm_1, arm_k].

w[i, 1] <- 0

delta[i, 1] <- 0

for (k in 2:na[i]) { # parameterize multi-arm trials using a trick to avoid dmnorm

delta[i, k] ~ dnorm(md[i, k], taud[i, k])

md[i, k] <- d[t[i, 1], t[i, k]] + sw[i, k]

taud[i, k] <- tau.d * 2 * (k - 1) / k

w[i, k] <- delta[i, k] - d[t[i, 1], t[i, k]]

sw[i, k] <- sum(w[i, 1:k-1]) / (k - 1)

}

}

# Relative effect matrix

d[1,1] <- 0

d[1,2] <- d.A.J + d.J.B

d[1,3] <- d.A.J + d.J.C

d[1,4] <- d.A.J + d.J.D

d[1,5] <- d.A.J + d.J.E

d[1,6] <- d.A.J + d.J.F

d[1,7] <- d.A.J + d.J.G

d[1,8] <- d.A.I

d[1,9] <- d.A.J

d[2,1] <- -d.A.J + -d.J.B

d[2,2] <- 0

d[2,3] <- -d.J.B + d.J.C

d[2,4] <- -d.J.B + d.J.D

d[2,5] <- -d.J.B + d.J.E

d[2,6] <- -d.J.B + d.J.F

d[2,7] <- -d.J.B + d.J.G

d[2,8] <- d.A.I + -d.A.J + -d.J.B

d[2,9] <- -d.J.B

d[3,1] <- -d.A.J + -d.J.C

d[3,2] <- d.J.B + -d.J.C

d[3,3] <- 0

d[3,4] <- -d.J.C + d.J.D

d[3,5] <- -d.J.C + d.J.E

d[3,6] <- -d.J.C + d.J.F

d[3,7] <- -d.J.C + d.J.G

d[3,8] <- d.A.I + -d.A.J + -d.J.C

d[3,9] <- -d.J.C

d[4,1] <- -d.A.J + -d.J.D

d[4,2] <- d.J.B + -d.J.D

d[4,3] <- d.J.C + -d.J.D

d[4,4] <- 0

d[4,5] <- -d.J.D + d.J.E

d[4,6] <- -d.J.D + d.J.F

d[4,7] <- -d.J.D + d.J.G

d[4,8] <- d.A.I + -d.A.J + -d.J.D

d[4,9] <- -d.J.D

d[5,1] <- -d.A.J + -d.J.E

d[5,2] <- d.J.B + -d.J.E

d[5,3] <- d.J.C + -d.J.E

d[5,4] <- d.J.D + -d.J.E

d[5,5] <- 0

d[5,6] <- -d.J.E + d.J.F

d[5,7] <- -d.J.E + d.J.G

d[5,8] <- d.A.I + -d.A.J + -d.J.E

d[5,9] <- -d.J.E

d[6,1] <- -d.A.J + -d.J.F

d[6,2] <- d.J.B + -d.J.F

d[6,3] <- d.J.C + -d.J.F

d[6,4] <- d.J.D + -d.J.F

d[6,5] <- d.J.E + -d.J.F

d[6,6] <- 0

d[6,7] <- -d.J.F + d.J.G

d[6,8] <- d.A.I + -d.A.J + -d.J.F

d[6,9] <- -d.J.F

d[7,1] <- -d.A.J + -d.J.G

d[7,2] <- d.J.B + -d.J.G

d[7,3] <- d.J.C + -d.J.G

d[7,4] <- d.J.D + -d.J.G

d[7,5] <- d.J.E + -d.J.G

d[7,6] <- d.J.F + -d.J.G

d[7,7] <- 0

d[7,8] <- d.A.I + -d.A.J + -d.J.G

d[7,9] <- -d.J.G

d[8,1] <- -d.A.I

d[8,2] <- -d.A.I + d.A.J + d.J.B

d[8,3] <- -d.A.I + d.A.J + d.J.C

d[8,4] <- -d.A.I + d.A.J + d.J.D

d[8,5] <- -d.A.I + d.A.J + d.J.E

d[8,6] <- -d.A.I + d.A.J + d.J.F

d[8,7] <- -d.A.I + d.A.J + d.J.G

d[8,8] <- 0

d[8,9] <- -d.A.I + d.A.J

d[9,1] <- -d.A.J

d[9,2] <- d.J.B

d[9,3] <- d.J.C

d[9,4] <- d.J.D

d[9,5] <- d.J.E

d[9,6] <- d.J.F

d[9,7] <- d.J.G

d[9,8] <- d.A.I + -d.A.J

d[9,9] <- 0

# Study baseline priors

for (i in 1:ns) {

mu[i] ~ dnorm(0, 1.649E-3)

}

# Variance prior

sd.d ~ dunif(0, 1.642E0)

tau.d <- pow(sd.d, -2)

# Effect parameter priors

d.A.I ~ dnorm(0, 1.649E-3)

d.A.J ~ dnorm(0, 1.649E-3)

d.J.B ~ dnorm(0, 1.649E-3)

d.J.C ~ dnorm(0, 1.649E-3)

d.J.D ~ dnorm(0, 1.649E-3)

d.J.E ~ dnorm(0, 1.649E-3)

d.J.F ~ dnorm(0, 1.649E-3)

d.J.G ~ dnorm(0, 1.649E-3)

}

list(

ns = 15,

na = c(2, 2, 2, 2, 2, 2, 2, 2, 2, 2, 2, 2, 2, 2, 2),

t = structure(.Data = c(9, 1, 9, 2, 9, 2, 9, 2, 9, 7, 9, 4, 9, 5, 9, 3, 9, 3, 9, 6, 9, 1, 9, 1, 9, 2, 9, 4, 1, 8), .Dim = c(15, 2)),

r = structure(.Data = c(15, 75, 20, 126, 8, 30, 6, 21, 10, 33, 33, 116, 47, 131, 59, 95, 60, 103, 12, 34, 7, 20, 54, 102, 2, 8, 2, 5, 29, 35), .Dim = c(15, 2)),

n = structure(.Data = c(110, 225, 170, 329, 18, 37, 65, 64, 14, 60, 153, 308, 131, 257, 326, 329, 210, 215, 42, 86, 36, 37, 170, 169, 22, 21, 12, 12, 54, 56), .Dim = c(15, 2))

)

list(

d.A.I = -1.290394357876605,

d.A.J = -1.6381181305415289,

d.J.B = 2.4921318423936745,

d.J.C = 0.9650809274537335,

d.J.D = 0.009539981905882344,

d.J.E = 0.5200064342202024,

d.J.F = -0.19318838723841486,

d.J.G = -0.37987536536902716,

mu = c(-2.3379639114537394, -1.3082533398433691, -0.16961455532471498, -0.8209675894132618, 2.049810297671325, -1.487664306925087, -0.20872338335181528, -1.574281331644692, -0.618632394908776, -2.8422629061945806, -2.694384789677512, -0.9275386515636926, -1.8097417407010268, 0.9320025250670783, 0.3343847619089417),

delta = structure(.Data = c(NA, 1.471601934716284, NA, 3.223625915689089, NA, 1.1065128547403589, NA, 1.7301200906619978, NA, -0.08677068117873266, NA, 0.515994564132054, NA, -0.2229858636150519, NA, 0.5675242556299338, NA, 0.8003199691775399, NA, 1.4010290222559696, NA, 1.983777427296864, NA, 0.7138667551172659, NA, -0.3817251798963017, NA, -0.8660772370066896, NA, -0.7722866916950812), .Dim = c(15, 2)),

sd.d = 1.5630418916528077

)

list(

d.A.I = 2.029856571327369,

d.A.J = -1.2999978641441123,

d.J.B = 1.6382355672519004,

d.J.C = 0.5168521759544673,

d.J.D = 1.1502347378888023,

d.J.E = 0.7832067655911201,

d.J.F = -0.3803158359097662,

d.J.G = -1.9922484147013853,

mu = c(-2.486349410652064, -1.5537398987207447, -0.05385410700541585, -0.963639777685007, 1.189760768075438, -2.0582893907752005, -0.26421465040337744, -1.3664867948181991, -0.9633870993496845, -1.9199235156584793, -2.4310276181219095, -0.1649654523628452, 0.3181108143360225, -0.7379451376402913, 0.7341790525954134),

delta = structure(.Data = c(NA, 0.8672675929428082, NA, 1.5675860719574786, NA, 3.985037031455154, NA, 0.12877690108697526, NA, 0.5367857939221914, NA, 0.7387256487814576, NA, 0.8076652353105792, NA, 1.533420176202411, NA, 0.5574555637078968, NA, -0.6723772335209479, NA, 0.0683425329721763, NA, 0.9012798020913428, NA, 3.5716152448216554, NA, -0.7549914664749608, NA, 1.5865571137510022), .Dim = c(15, 2)),

sd.d = 0.8560438390708119

)

list(

d.A.I = 1.3063490307237728,

d.A.J = -1.1800114770704402,

d.J.B = 2.392704853576837,

d.J.C = 0.8458548888442403,

d.J.D = 0.6794606265769736,

d.J.E = 0.5477257169925102,

d.J.F = 1.6932716181438698,

d.J.G = -0.42864318238011195,

mu = c(-2.8357585565913532, -2.514180787428919, 0.3120642518545162, -2.2317284467150618, 0.544211669008132, -0.8028229750059621, -0.8392447862185731, -1.7895403890429389, -0.27154351101112006, 0.23418849539221787, -0.2736201602471233, -0.6944297004599091, -1.0031922344823643, -0.7713236239627049, -0.600861563691754),

delta = structure(.Data = c(NA, 0.7071758559553587, NA, 0.8163987197877556, NA, 2.070663927116905, NA, 2.7592447237600224, NA, 0.7981706950268816, NA, -0.15131207775550515, NA, 1.106233600385889, NA, 0.07652237463270273, NA, 0.26171451477103247, NA, -2.182670076229311, NA, 1.6471107100151696, NA, 1.509246021271828, NA, 2.5477283062266607, NA, 1.202344650512209, NA, 0.5881845027971222), .Dim = c(15, 2)),

sd.d = 0.9951720401797386

)

list(

d.A.I = 1.6708643507638952,

d.A.J = -1.2472720539830557,

d.J.B = 2.0138345709696366,

d.J.C = 0.417604752181385,

d.J.D = 1.4209744813843872,

d.J.E = 0.2228208422499619,

d.J.F = 2.204315489589043,

d.J.G = -0.0022933373166389437,

mu = c(-1.8712017857275227, -0.9401158992348331, 0.43333151202278025, -3.928627836540578, -0.9290033549805283, -1.2053976029338611, -0.6542013690906445, -1.7753770614972721, -0.7195323493522053, -1.986307201527738, -2.183522341847879, -0.7594193191724805, -1.9758903046115333, -3.9405412876145665, -0.021247922391979757),

delta = structure(.Data = c(NA, 0.6200623555961928, NA, 1.4193911889912572, NA, 3.078406521054277, NA, 1.8489894564735578, NA, 1.950174372937103, NA, 0.8615812377866685, NA, 2.0574896675161827, NA, 1.475077046223479, NA, 0.7697318145832069, NA, 0.7116747725354413, NA, 3.2735591807202766, NA, 1.3284620701466396, NA, -1.165311408073145, NA, -2.134761656695535, NA, 0.4763673300185296), .Dim = c(15, 2)),

sd.d = 0.13629591383979847

)

modelCheck('Consistency Model.cons.model')

modelData('Consistency Model.cons.data')

modelCompile(4)

modelInits('Consistency Model.cons.inits1', 1)

modelInits('Consistency Model.cons.inits2', 2)

modelInits('Consistency Model.cons.inits3', 3)

modelInits('Consistency Model.cons.inits4', 4)

modelSetAP('slice', 20000)

modelUpdate(20000)

samplesSet('d.A.I')

samplesSet('d.A.J')

samplesSet('d.J.B')

samplesSet('d.J.C')

samplesSet('d.J.D')

samplesSet('d.J.E')

samplesSet('d.J.F')

samplesSet('d.J.G')

samplesSet('sd.d')

modelUpdate(400000)

samplesCoda('*', 'Consistency Model.cons')
